# The predictive potential of different molecular markers linked to amikacin susceptibility phenotypes in *Pseudomonas aeruginosa*

**DOI:** 10.1371/journal.pone.0267396

**Published:** 2022-04-25

**Authors:** Wedad M. Nageeb, Helal F. Hetta

**Affiliations:** 1 Medical Microbiology and Immunology Department, Faculty of Medicine, Suez Canal University, Ismailia, Egypt; 2 Medical Microbiology and Immunology Department, Faculty of Medicine, Assiut University, Assiut, Egypt; Qassim University, College of Public Health and Informatics, SAUDI ARABIA

## Abstract

Informed antibiotic prescription offers a practical solution to antibiotic resistance problem. With the increasing affordability of different sequencing technologies, molecular-based resistance prediction would direct proper antibiotic selection and preserve available agents. Amikacin is a broad-spectrum aminoglycoside exhibiting higher clinical efficacy and less resistance rates in *Ps*. *aeruginosa* due to its structural nature and its ability to achieve higher serum concentrations at lower therapeutic doses. This study examines the predictive potential of molecular markers underlying amikacin susceptibility phenotypes in order to provide improved diagnostic panels. Using a predictive model, genes and variants underlying amikacin resistance have been statistically and functionally explored in a large comprehensive and diverse set of *Ps*. *aeruginosa* completely sequenced genomes. Different genes and variants have been examined for their predictive potential and functional correlation to amikacin susceptibility phenotypes. Three predictive sets of molecular markers have been identified and can be used in a complementary manner, offering promising molecular diagnostics. *arm*R, *nal*C, *nal*D, *mex*R, *mex*Z, *amp*R, *rmt*D, *nal*DSer32Asn, *fus*A1Y552C, *fus*A1D588G, *arn*AA170T, and *arn*DG206C have been identified as the best amikacin resistance predictors in *Ps*. *aeruginosa* while *fao*AT385A, *nuo*GA890T, *nuo*GA574T, *lpt*AT55A, *lpt*AR62S, *pst*BR87C, *gid*BE126G, *gid*BQ28K, *amg*SE108Q, and *rpl*YQ41L have been identified as the best amikacin susceptibility predictors. Combining different measures of predictive performance together with further functional analysis can help design new and more informative molecular diagnostic panels. This would greatly inform and direct point of care diagnosis and prescription, which would consequently preserve amikacin functionality and usefulness.

## Introduction

Although great advances have been achieved in diagnostic technologies, empiric antimicrobial prescription is still widely used to deal with critical infections [1, 2]. This consequently results in the overuse of our small inventory of effective and last line antimicrobial agents [3, 4]. That situation has resulted in the aggravation of antibiotic resistance problem by driving the emergence and spread of multi-drug resistant organisms. The gap that currently exists between the traditional microbiology workflow and the need for more rapid results, especially in some critical conditions, has led to the current problem of overtreatment. Improved molecular diagnostics would overcome such a situation. A better diagnostic would provide reliable information on susceptibility to antimicrobial agents, which would consequently influence the best choice of treatment.

Accurate diagnosis is an essential step for the successful management of any health problem. Although higher test accuracy is often used as an important indicator for the usefulness of the test, this does not necessarily indicate that tests with higher accuracy often lead to improved health outcomes and are considered the tests of choice in clinical practice [5]. Acceptable standards in positive and negative predictive values that can translate into changes in patient management are still lacking [6]. This consequently necessitates considering all parameters used to assess the accuracy of the diagnostic test in a setting-relevant basis and according to the condition being investigated.

With the recent advances in different diagnostics technologies, there has been a growing interest in developing new and rapid diagnostics for bacterial resistance [7, 8]. Many newer technologies have been used, including Matrix-Assisted Laser Desorption/Ionization-Time Of Flight Mass Spectrometry MALDI-TOF MS, fluorescent live/dead staining, infrared spectroscopy, microbial cell weighing by vibrating cantilevers, magnetic bead spin, and microdroplets, among others [9]. In addition to the accelerated phenotypic systems, different molecular platforms have also been used for rapid bacterial identification and antimicrobial susceptibility testing (ID/AST) testing [10–15]. With the increasing use and affordability of different sequencing platforms, sequencing-based resistance prediction is now expected to offer a better diagnostic alternative for antimicrobial resistance [16]. It would enable the detection of genetic markers underlying specific risky profiles and also help in the detection of genetic markers predictive of different antimicrobial susceptibility phenotypes.

Amikacin is a semisynthetic aminoglycoside antibiotic with a broad antimicrobial spectrum. According to the British National Formulary (BNF) [17], aminoglycosides are still widely used in the treatment of serious infections caused by multiple Gram negative organisms, including *Ps*. *aeruginosa*. Amikacin is considered more stable to enzyme inactivation than gentamycin and is used in the treatment of serious infections caused by gentamycin-resistant organisms, including biliary infections, septicemia, and endocarditis. Aminoglycosides have been considered as a vital component in antipseudomonal chemotherapy, including combined therapy regimens, particularly pulmonary infections in cystic fibrosis patients [18]. Amikacin has the broadest spectrum of activity among the aminoglycosides group of antibiotics and is considered a good treatment candidate to strains showing multiple resistance to other aminoglycosides [19]. Both gentamycin and amikacin have been used in the treatment of urinary tract infections caused by *Ps*. *aeruginosa* with amikacin achieving better peak serum concentrations at lower therapeutic doses [20]. Amikacin is also known to exhibit less resistance rates due to its structural nature [21]. In addition, amikacin shows activity against gentamycin resistant strains and can achieve high blood levels making it clinically effective in the treatment of *Pseudomonas*-associated pulmonary infections complicating cystic fibrosis [22]. Recent evidence also shows that amikacin can achieve high *in vitro* potency against *Ps*. *aeruginosa* respiratory and blood isolates [23]. Kim *et al*., (2018) have also demonstrated declining trends of gentamycin and amikacin resistance in *Ps*. *aeruginosa* according to data from the Korean Nationwide Surveillance of Antimicrobial Resistance (KONSAR) program, which was attributable to decreased aminoglycosides consumption levels [24]. This finding indicates that the rational use of this antibiotic would alleviate the selective pressure on the agent and consequently decrease emergence of resistance. In order to preserve such valuable therapeutic agent and to avoid failure of therapy, it is important to prescribe it wisely. Rational use can be achieved through wiser prescription which can be achieved by using rapid resistance predictors. From that perspective, this study has evaluated the chromosomal genetic variants capable of predicting amikacin susceptibility highlighting their potential use as diagnostic markers. A group of genes and variants previously identified in the literature as related to amikacin resistance were reviewed and assessed in this work for their predictive potential. In addition, new variants of the same resistance-related genes were also evaluated for their diagnostic potential. The approach implemented in this study has combined different measures of diagnostic accuracy in a complementary way in addition to functional evidence to investigate the candidate molecular markers. Although amikacin is known to be stable to the action of aminoglycoside inactivating enzymes, both aminoglycoside 6’-N-acetyltransferase Ib (AAC(6’)-Ib) [25] and aminoglycoside nucleotidyl transferase (*ant*4’-IIb) [26] have been reported to affect amikacin activity in *Ps*. *aeruginosa*. In addition, other inactivating enzymes including the 16S rRNA methylase *rmt*A [27] and *rmt*D [28] have also been identified to confer amikacin resistance in *Ps*. *aeruginosa*. So, these enzymes have also been explored in the current set of studied sequences in order to investigate their importance and relative contribution to amikacin resistance.

## Materials and methods

In this study, completely sequenced genomes with their laboratory-measured phenotypic data for amikacin were downloaded from the Patric database [29]. The list of genome sequences included in the study with corresponding amikacin MICs is shown in S1 Table. The analysis included a total of 528 *Ps*. *aeruginosa* genome sequences from Patric database. The sequences used have been previously assessed for their diverse representation [30]. Breakpoints for analysis of susceptibility and resistance were defined according to the latest EUCAST recommendations [31]. Based on breakpoints definition, the available sequences studied included a total of 109 resistant isolates and 419 susceptible isolates. The MIC values were distributed over the ranges of 0.12, 0.25, 0.5, 1, 2, 4, 8, 16, 32, 64, and 128. Literature review to extract chromosomal genes and gene variants related to aminoglycoside resistance was carried out on each of PMC PubMed, ACADEMIC SEARCH COMPLETE (EBSCO host), and ScienceDirect using search criteria: "*Pseudomonas aeruginosa*"[title/abstract] AND "aminoglycosides resistance"[title/abstract]. Genes and variants identified from the literature are listed in S2 Table. Predictive values for the whole identified set consisting of 55 molecular markers have been examined, and those showing higher predictive performance to amikacin have been further functionally analyzed. The genes extracted from the literature and investigated in the current study included *mex*R, *mex*S, *nal*C, *nal*D, *nfx*B, *amp*R, *gid*B, *amg*S, *pmr*A, *pmr*B, *fus*A1, *rpl*Y, *arn*A, *arn*D, *lpt*A, *fao*A, *pst*B, *pho*P, *pho*Q and *nuo*G.

The set consisting of 55 chromosomal elements as well as 3 aminoglycoside inactivating enzymes that have been reported to exhibit activity against amikacin have been used as an input to construct a predictive model for amikacin susceptibility. Stepwise multiple regression was conducted to examine the extent of variance in amikacin susceptibility phenotype as explained by different molecular markers being investigated giving an indication about the relative contribution of each marker to the phenotype. This has been implemented using SPSS (SPSS.V21) [32].

The sequence of each of the chromosomal genes was extracted by searching the *Pseudomonas* genome database [33] available at https://www.pseudomonas.com/ and the NCBI database at https://www.ncbi.nlm.nih.gov/gene using the known gene identifier ID and/or the gene name. The available gene sequence was downloaded from *Pseudomonas* genome database or from NCBI by constructing a FASTA file using the sequence available on either of the two databases. In addition, the sequences of the genes coding for inactivating enzymes; *aac*(6’)-Ib, *ant*4’-IIb, *rmt*A, and *rmt*D has also been extracted from NCBI database and later BLASTed to identify their frequency of occurrence in the studied set of sequences. The NCBI BLAST+ BLASTN tool available at https://usegalaxy.org/ was used to search nucleotide database with nucleotide query sequence(s) (Galaxy Version 0.3.3) [34]. The nucleotide query sequence of the gene sequences extracted above was used to search the constructed nucleotide BLAST database using the megaBLAST option and the default Set expectation value cutoff at 0.001.

The aligned part of both subject and query sequences in FASTA format was visualized and explored using MEGA 7 software [35]. All genes included in the current analyses showed > 80% percentage identity and >95% query coverage. Genes of interest were manually explored in detail to extract variants of interest (previously reported in the literature) as well as novel variants (amino acids or nucleotide changes showing specific differential patterns of distribution). A matrix showing the distribution of each of the variants of interest was generated and was then used to conduct further analyses. The functional effect of the identified gene variants was then tested. To do so, protein sequence information of the genes studied showing the evaluated variants were retrieved from *Pseudomonas* genome database [36]. Possible functional effect of amino acid changes identified in the variants of interest was evaluated using PROVEAN (Protein Variation Effect Analyzer) available at http://provean.jcvi.org./index.php [37] and I-Mutant v2.0 (Predictor of Protein Stability Changes upon Mutations) available at http://gpcr.biocomp.unibo.it/cgi/predictors/I-Mutant2.0/I-Mutant2.0.cgi [38].

The distribution of the mutations was then tested for its correlational pattern with phenotype and different measures of diagnostic accuracy were evaluated to test their diagnostic benefit and potential use as molecular predictive markers.

To do so, the variant distribution matrix generated was used to construct a 2*2 contingency table for each variant in relation to resistance/susceptibility phenotype. “Cross Tab” function was used to generate these contingency tables using SPSS (SPSS.V21) [32] with checking all the options to calculate chisquare test for independence or Fischer exact, significance value, phi coefficient, cramer V and likelihood ratios.

Parameters of performance for each single variant including, Sensitivity, Specificity, Negative Predictive Value (NPV), Positive Predictive Value (PPV), Likelihood Ratio (LR), Likelihood Ratio positive (LHR+), Diagnostic Odds Ratio (DOR), and diagnostic accuracy for each contingency table were calculated according to the following equations; Sensitivity = TP/TP+FN, Specificity = TN/TN+FP, PPV = TP/TP+FP, NPV = TN/TN+FN, LHR+ = sensitivity / (1-specificity), DOR = LHR+/ LHR- or DOR = sensitivity* specificity/ [(1-sensitivity)*(1-specificity)]. Diagnostic accuracy = (TP+TN)/(TP+TN+FP+FN).

In order to test for the possible functional effect of the prioritized markers and consequently their possible practical significance, available 3D protein structures for some of the identified proteins at the protein data bank [39] were downloaded at https://www.rcsb.org/. The following PDB IDs were evaluated as macromolecule receptors; 5H9T, 4FN5, 2D3T, 2R1A, 1JSX. Binding of amikacin as a ligand to these macromolecules has been tested and compared in both variant and non-substituted protein. Amikacin molecule structure was retrieved from pubchem at https://pubchem.ncbi.nlm.nih.gov/. The 3D conformer PubChem CID 37768 SDF file for amikacin was used. The PDB file for each of the tested macromolecules and ligand file were uploaded and the interaction of amikacin with each of the evaluated proteins was tested using in-silico molecular docking by running autodock vina [40] using PyRx software [41]. Docking results were visualized and analyzed using BIOVIA Discovery Studio Visualizer v21.1.0.20298, Release 2020, San Diego: Dassault Systèmes.

## Results

The study builds a predictive model to interpret variability in amikacin MIC based on a group of chromosomal and also acquired elements. Fifty-five analyzed predictor chromosomal gene elements have been used to establish a predictor model. The studied elements have also been statistically and functionally analyzed to identify a useful group of potential diagnostic markers. Stepwise multiple regression analysis was used to assess the ability of variants showing higher correlations with phenotype to predict the level of amikacin MIC. Excluding variants showing multicollinearity, the most important predictors in the predictive model included *pst*BE89Q, *nal*Dser32Asn, *fus*A1Y552C, *pmr*BLeu323His, *amp*R, and *amp*RE114A. In the proposed model, only 12.2% of variance in the dependent variable (Amikacin MIC) was explained by the above 6 predictors in the model (*p*<0.0005). When inactivating enzymes were included in the model input, 22.6% of variance in amikacin MIC could be explained by *rmt*D, *pst*BE89Q, *pmr*BLeu323His, *amp*R, *fus*A1Y552C, and *nal*Dser32Asn. When the statistical effect of overlapping variables was excluded, variables making significant unique contribution to the prediction of the dependent variable (Amikacin MIC) included in order of importance: *rmtD* (*beta* = -0.333, *p*<0.0005), *pst*BE89Q (*beta* = -0.268, *p*<0.0005), *pmr*BLeu323His (*beta* = -0.144, *p*<0.0005), *amp R* (*beta* = 0.137, *p*<0.0005), *fus*A1Y552C (*beta* = - 0.134, *p* = 0.001),and *nal*Dser32Asn (*beta* = -0.126, *p* = 0.001). When the other variants were re-included as predictors for a better model, 26.9% of variance in Amikacin MIC (*p*<0.0005) could be explained by 39 markers including *amp*RA51T, *pst*BR87C, *amp*RD135N, *nal*DD187H, *rpl*YQ41L, *mex*R, *rmt*D, *fao*AT385A, *nal*Dl153Q, *nuo*GA890T, *pst*BE89Q, *amp*R, *lpt*AT55A, *nal*CE153Q, *lpt*AR62S, *fus*A1Y552C, *nal*Dser32Asn, *aac*6, *pmr*BA248V, *pmr*ALeu71Arg, *nuo*GS468A, *nal*CG71E, *fus*A1D588G, *amp*RE114A, *pmr*BALA4Thr, *nal*CA186T, *amp*RG283E, *nal*CS46A, *rpl*YAla123Ser, *pho*QY85F, *nal*D, *gid*BE126G, *nal*CS209R, *nuo*GA574T, *mex*Z, *amp*RM288R, *mex*RR79S, *nal*C gene, *rmt*A.

Elements prioritized from the predictive model and those showing the highest predictive values have been then functionally analyzed to add to the evidence of their potential to act as useful diagnostic predictors. Analysis of molecular markers related to amikacin resistance showed that variants in the genes related to cell membrane proteins and those related to cell division were the most important in accounting for resistance showing the highest predictive values and highest performance among the complete set of 55 examined chromosomal elements. These markers can be used to rule in amikacin resistance showing higher specificity. The variants identified included *nal*DSer32Asn, *fus*A1Y552C, *fus*A1D588G, *arn*AA170T, *arn*DG206C as markers of resistance. In addition, the 16S rRNA methylases *rmt*A and *rmt*D also showed high specificity. The predictive values and other measures of performance related to this group of markers are shown in Table 1.

**Table 1:** Measures of diagnostic accuracy for variants related to amikacin resistance.

Molecular marker	Specificity %	PPV %	NPV %	LR	LR+	DOR	Accuracy
*nal*DSer32Asn	99.3	62.5	80	6.7	6.57	6.84	0.80
*fus*A1Y552C	99.5	71.4	80	8.48	9.2	9.60	0.80
*fus*A1D588G	100	100	80	12.7	37	38.38	0.80
*arn*AA170T	98.1	65.2	81.4	22.6	7.26	8.27	0.81
*arn*DG206C	97.9	60.9	81.2	18.6	6.1	6.84	0.80
*rmtA*	99.8	83.3	80.1	11	23	20.86	0.80
*rmtD*	100	100	80.1	15.96	--	--	0.80

Another group of efflux-pump related-genes has also been identified as potential diagnostic markers which presence does not guarantee amikacin susceptibility, but its absence can be used to rule out susceptibility and hence predict amikacin resistance with higher confidence. These markers demonstrate high sensitivity and higher positive predictive values and thus can be used as screening markers. These are shown in Table 2 with their corresponding measures of performance.

**Table 2:** Measures of diagnostic accuracy for genes related to amikacin resistance.

Molecular marker	Sensitivity %	PPV %	LR	LR+	DOR	Accuracy
*arm*R	94	80.2	3	1.06	1.94	0.77
*nal*C	95.2	80.3	3.9	1.06	2.23	0.78
*nal*D	92.8	80.2	2.4	1.05	1.74	0.76
*mex*R	99	79.3	0	0.99	0.89	0.79
*mex*Z	94.7	80.4	4.2	1.06	2.21	0.77
*amp*R	95.5	80.3	4.4	1.06	2.38	0.78

Another important finding is the observation of a group of molecular markers showing high specificity and high positive predictive values to amikacin susceptibility and these can be considered as molecular susceptibility markers used to rule in the diagnosis of amikacin susceptibility. These include *fao*AT385A, *nuo*GA890T, *nuo*GA574T, *lpt*AT55A, *lpt*AR62S, *pst*B R87C, *gid*BE126G, *gid*BQ28K, *amg*SE108Q, and *rpl*YQ41L. The predictive values and other measures of performance related to this group of markers for amikacin are shown in Table 3.

**Table 3:** Measures of diagnostic accuracy for variants related to amikacin susceptibility.

Molecular marker	Specificity %	PPV %	LR	LR+	DOR	Accuracy
*fao*AT385A	100	100	3.7	19	19.34	0.22
*nuo*GA890T	100	100	7	36	37.31	0.23
*nuo*GA574T	99.1	96	6	6.33	6.66	0.25
*lpt*AT55A	95.4	86.1	1.2	1.61	1.66	0.26
*lpt*AR62S	98.2	88.9	1.2	2.11	2.16	0.23
*pst*BR87C	100	100	1	50	52.58	0.21
*gid*BE126G	100	100	2.8	14	14.18	0.22
*gid*BQ28K	100	100	3.7	19	19.35	0.22
*amg*SE108Q	100	100	3.7	19	19.35	0.22
*rpl*YQ41L	100	100	1.9	10	10.09	0.21

From the current analysis, it appears that aminoglycoside inactivating enzymes are not significant contributors to amikacin resistance. Although *rmt*D appears to be an important predictor for amikacin resistance contributing to 10% of variance in amikacin MIC as proposed by the predictive model and also showing 100% specificity and 100% PPV, it was infrequently encountered being identified only in 5 sequences showing MIC of 128 together with *rmt*A carried among the same sequences. On the other hand, *AAC(6’)-Ib* has been identified only in 3 amikacin resistant sequences and was also identified in another 10 amikacin susceptible sequences with an MIC of 8. It also did not significantly contribute to the predictive model showing lower predictive values.

To complement statistical evidence together with functional evidence about the utility of the identified markers, in silico functional prediction together with variants docking have been carried out. The predicted functional effects for the identified variants as tested using PROVEAN and I-Mutant are shown in Table 4.

**Table 4:** Predicted functional effects of variants identified as potential diagnostic markers.

Molecular marker/ variant	PROVEAN prediction		I-Mutant prediction	
	Predicted effect (cutoff)	PROVEAN score ^a^	Protein Stability	Reliability Index ^b^
*nal*DSer32Asn	Deleterious (-2.5)	-2.500	Decreased stability	2
*fus*A1Y552C	Deleterious (-2.5)	-7.256	Large decrease in stability	0
*fus*A1D588G	Deleterious (-2.5)	-6.198	Large decrease in stability	9
*arn*AA170T	Deleterious (-1.3)	-2.065	Decreased stability	4
*arn*DG206C	Deleterious (-2.5)	-8.374	Large decrease in stability	9
*fao*AT385A	Deleterious (-2.5)	-3.272	Large decrease in stability	8
*nuo*GA890T	Neutral (-2.5)	-0.131	Large decrease in stability	9
*nuo*GA574T	Deleterious (-1.3)	-1.940	Decreased stability	4
*lpt*AT55A	Neutral (-2.5)	0.852	Large decrease in stability	9
*lpt*AR62S	Deleterious (-2.5)	-3.392	Large decrease in stability	9
*pst*BR87C	Deleterious (-2.5)	-6.874	Large decrease in stability	8
*gid*BE126G	Deleterious (-2.5)	-2.779	Large decrease in stability	9
*gid*BQ28K	Deleterious (-1.3)	-1.442	Decreased stability	3
*amg*SE108Q	Deleterious (-1.3)	-1.774	Increased stability	5
*rpl*YQ41L	Deleterious (-2.5)	-3.098	Decreased stability	6

^a^Variants showing PROVEAN score less than the used cutoff are predicted as deleterious.

^b^Reliability Index shows the degree of reliability of the predicted effect on protein function stability; the higher the number, the more reliable the prediction is.

**Fig 1:**
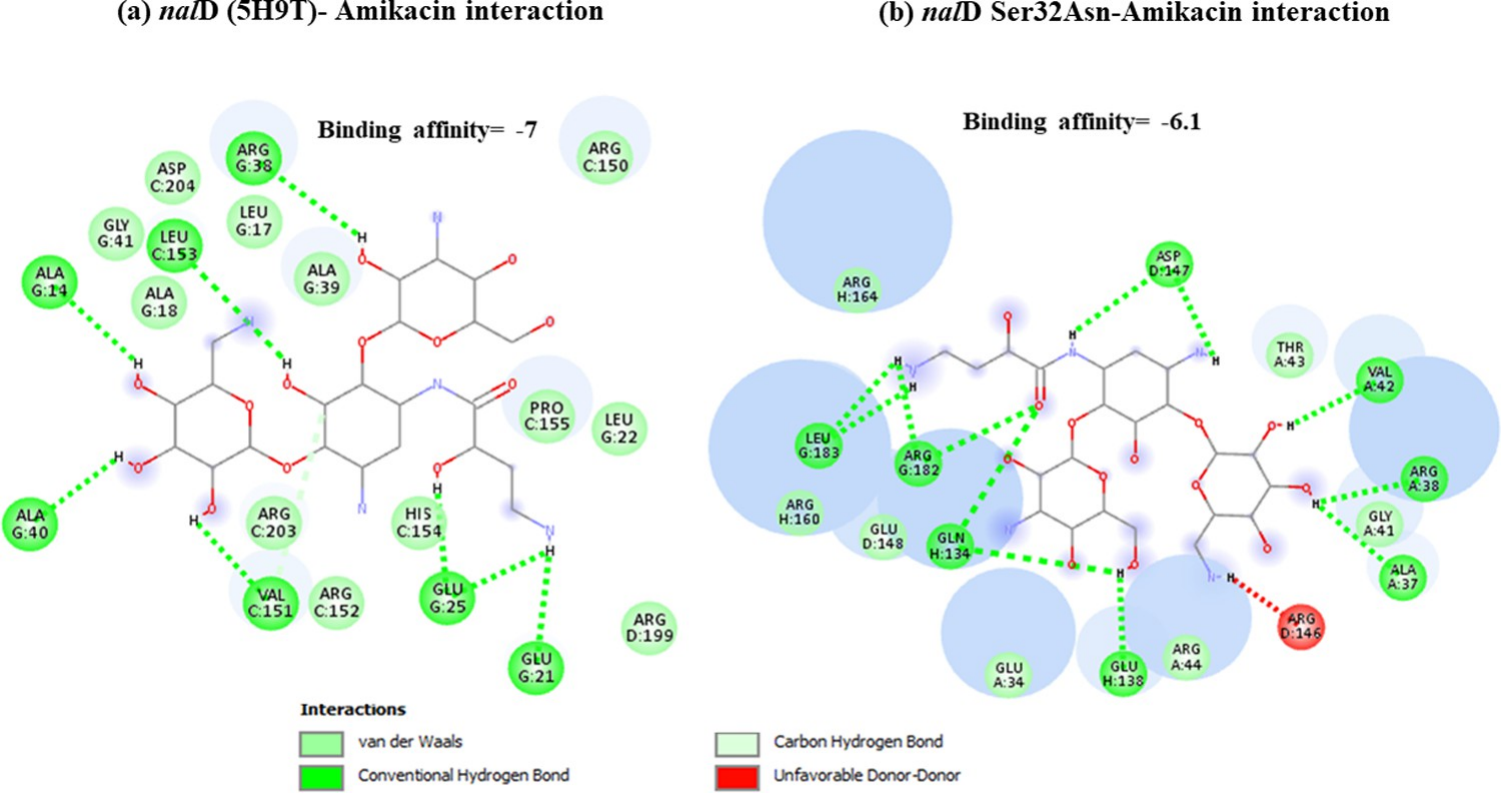
Comparison of amikacin interaction with *nal*D (5H9T) and *nal*DSer32Asn. (a) *nal*D (5H9T)- Amikacin interaction showed binding affinity of -7; (b) Amikacin interaction with *nal*DSer32Asn mutant showed lower binding affinity of– 6.1.

**Fig 2:**
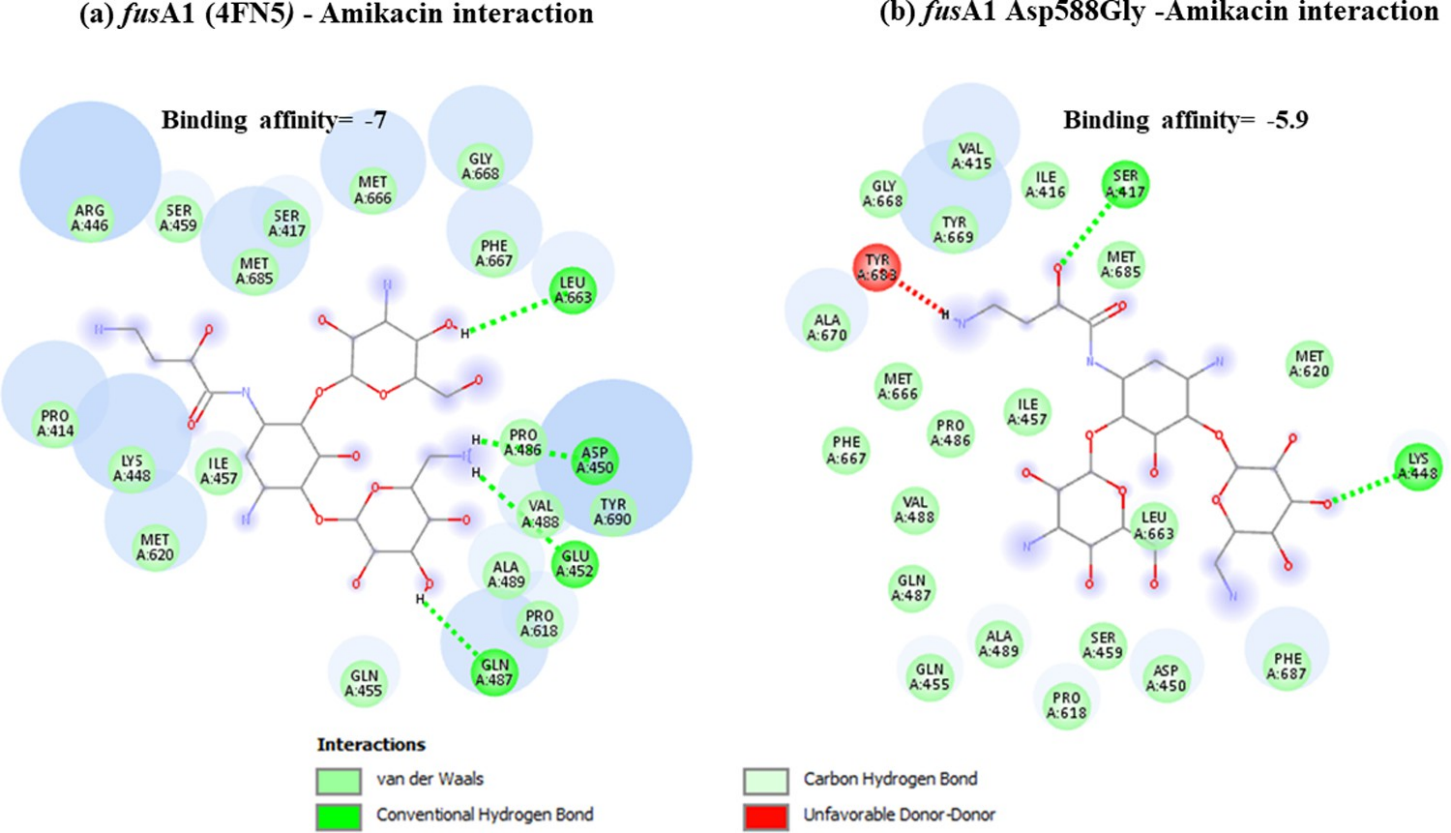
Comparison of amikacin interaction with *fus*A1 (4FN5*)* and *fus*A1Asp588Gly. (a) *fusA1 (4FN5)—*Amikacin interaction showed binding affinity of -7; (b) Amikacin interaction with *fus*A1Asp588Gly mutant showed lower binding affinity of– 5.9.

**Fig 3:**
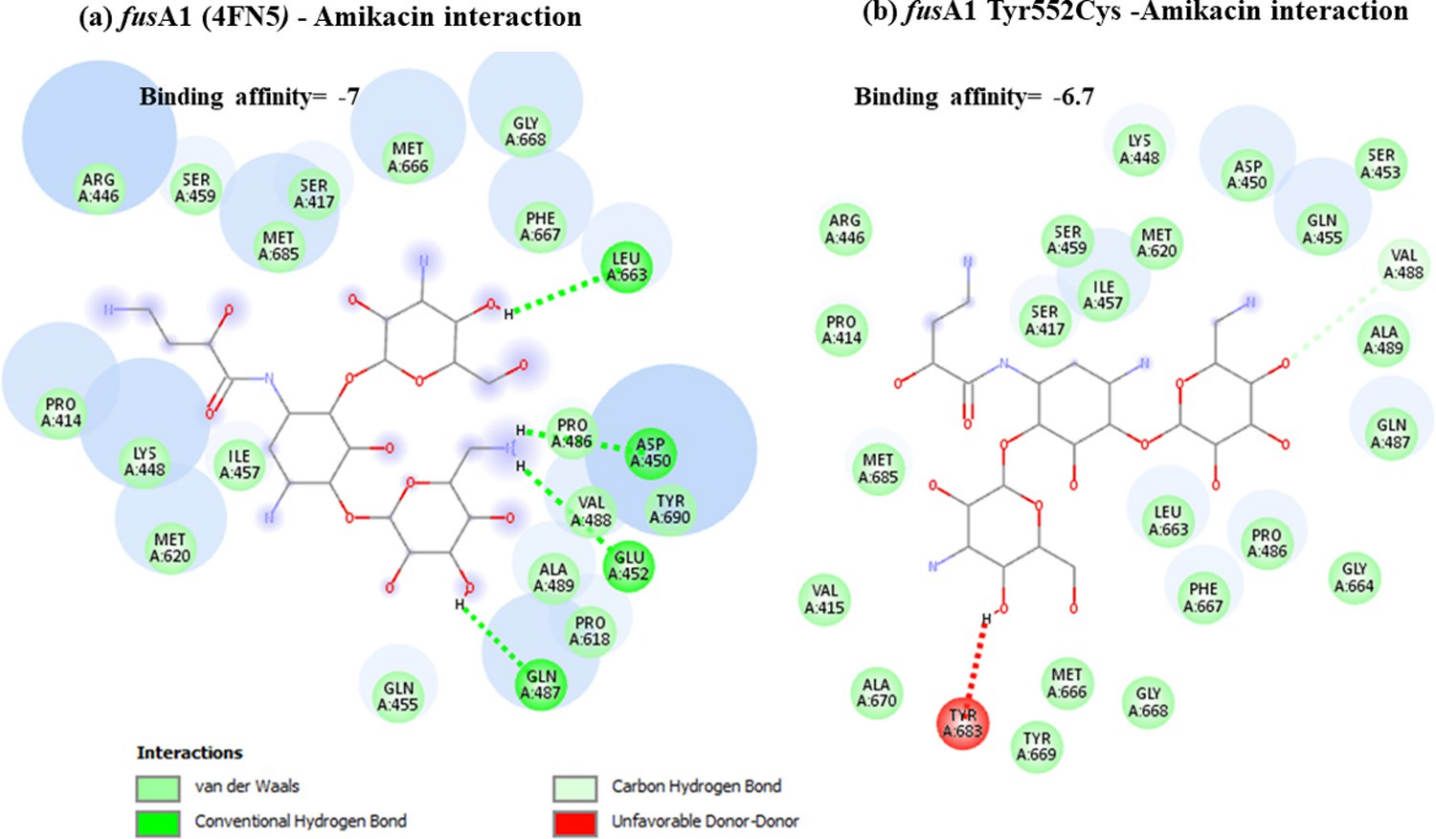
Comparison of amikacin interaction with *fus*A1 (4FN5*)* and *fus*A1Tyr552Cys. (a) *fus*A1 (4FN5*)*- Amikacin interaction showed binding affinity of -7; (b) Amikacin interaction with *fus*A1Tyr552Cys mutant showed lower binding affinity of– 6.7.

**Fig 4:**
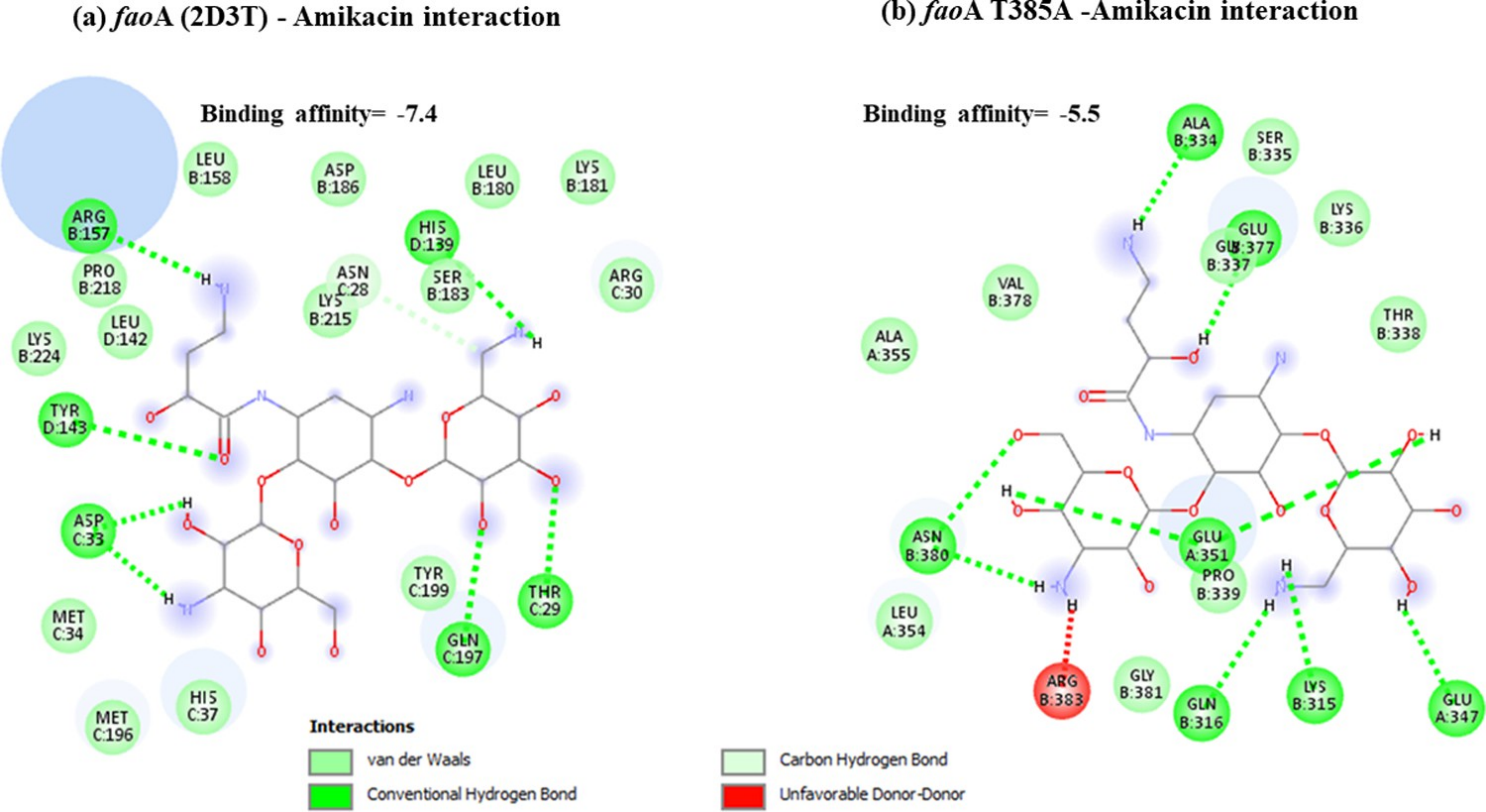
Comparison of amikacin interaction with *fao*A (2D3T) and *fao*AT385A. (a) *fao*A (2D3T)*—*Amikacin interaction showed binding affinity of -7.4; (b) Amikacin interaction with *fao*AT385A mutant showed lower binding affinity of– 5.5.

**Fig 5:**
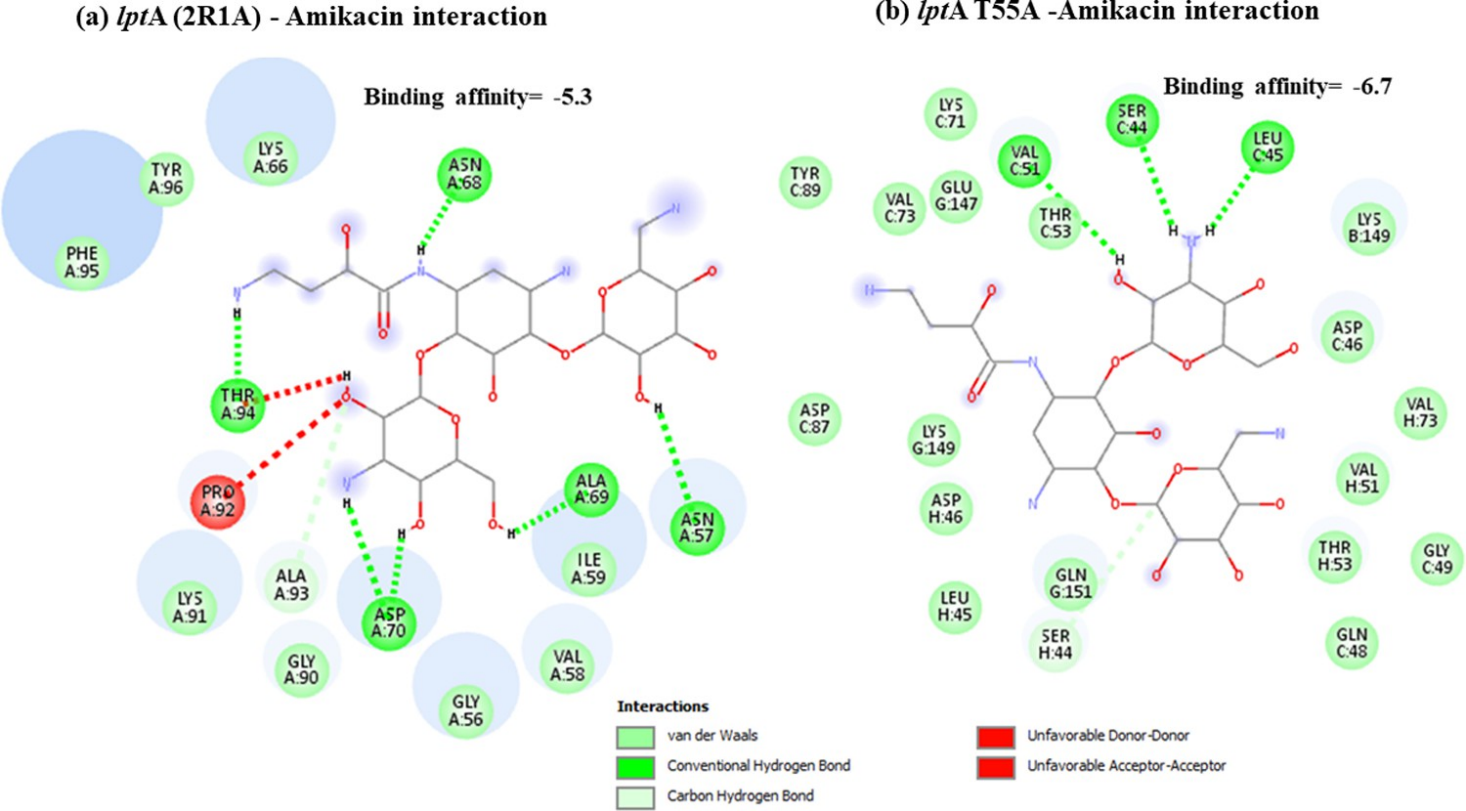
Comparison of amikacin interaction with *lpt*A (2R1A) and *lpt*AT55A. (a) *lpt*A (2R1A)—Amikacin interaction showed binding affinity of -5.3; (b) Amikacin interaction with *lpt*AT55A mutant showed higher binding affinity of– 6.7.

**Fig 6:**
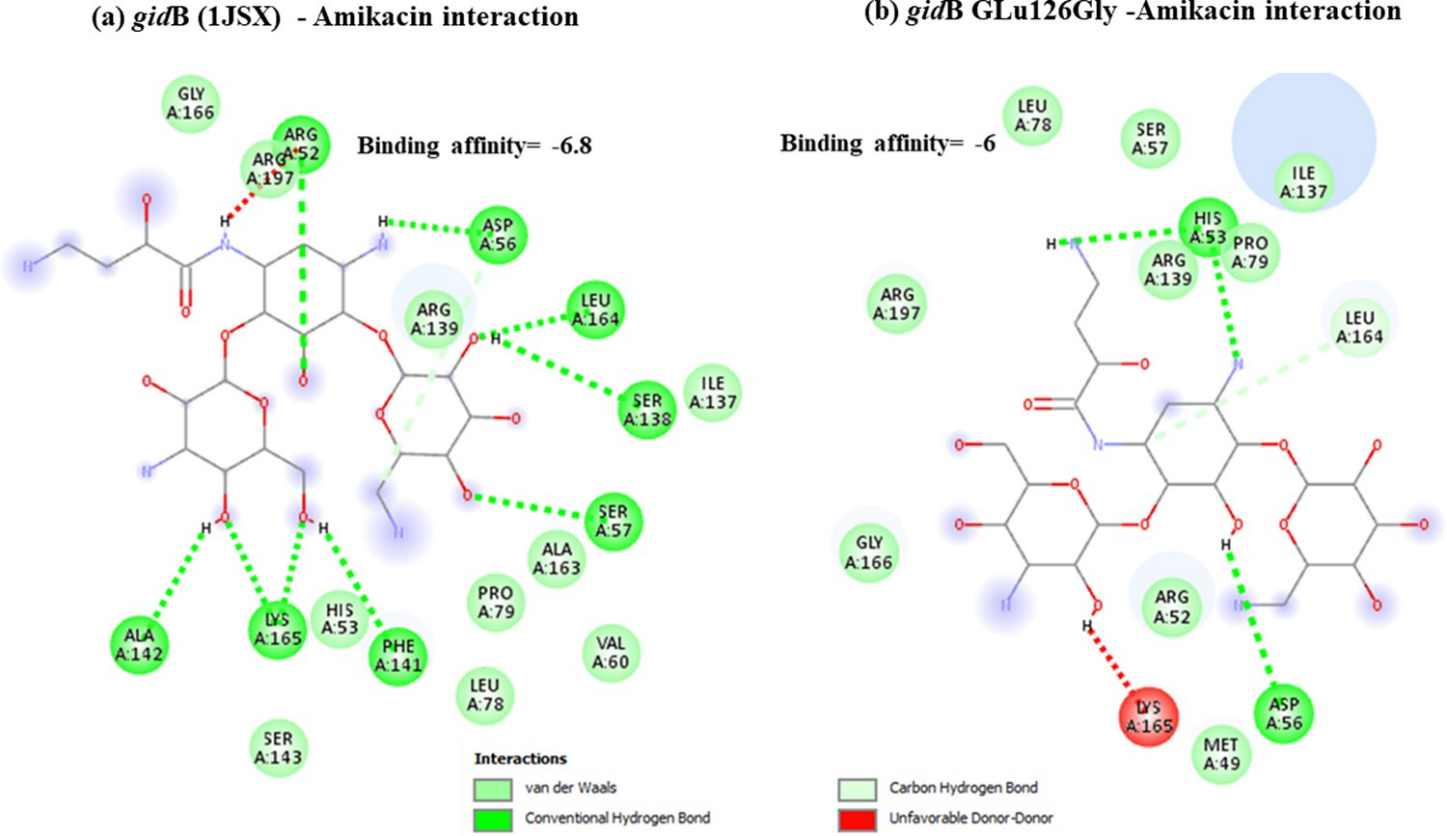
Comparison of amikacin interaction with *gid*B (1JSX) and *gid*BGLu126Gly. (a) *gid*B (1JSX)—Amikacin interaction showed binding affinity of -6.8; (b) Amikacin interaction with *gid*BGLu126Gly mutant showed lower binding affinity of– 6.

Docking results have shown that *nal*DSer32Asn mutant had lower binding to amikacin when compared to non-substituted protein with unfavorable state of donor-donor interaction observed at ARG-146 (Fig 1). This might explain the deleterious effect predicted for this mutant resulting in decreased stability. Lower binding affinities have also been observed for both types of examined *fus*A1 mutants (Figs 2 and 3). Both *fus*A1Asp588Gly and *fus*A1Tyr552Cys have shown an unfavorable state of donor-donor interaction at TYR 683, and these changes might also explain the deleterious effect and the largely decreased stability predicted for these mutants. Similarly, *fao*A T385A mutant has shown decreased amikacin binding affinity when compared to non-substituted protein (Fig 4), which may also confirm the prediction of its deleterious effect and may account for the predicted large decrease in stability. An unfavorable state of donor donor interaction was observed at ARG-383 (Fig 4). *gid*BGLu126Gly -amikacin interaction has also shown a lower binding affinity (Fig 6) with unfavorable donor-donor interaction at LYS-165. On the other hand, docking of *lpt*A protein (2R1A) with amikacin revealed increased affinities of the *lpt*AT55A variant when compared to non-mutant protein (Fig 5). Two unfavorable states of interactions have been observed at THR-94 and PRO-92. Docking results showing binding of amikacin to different variant and non-substituted proteins are shown in Figs 1–6.

## Discussion

High specificity is usually considered an important parameter to rule in any diagnosis. A group of markers showing high specificity and high LR+ to the phenotype under assessment can be used as molecular markers to rule in the diagnosis under consideration [42]. The diagnostic value of this type of markers is higher when the marker combines high specificity and high LR+ with high DOR, high accuracy, and higher PPV towards the same phenotype. This has been demonstrated in the current group of studied molecular markers in two sets of markers. The first set which can be used to rule in amikacin resistance included *nal*D Ser32Asn, *fus*A1Y552C, *fus*A1D588G, *arn*A A170T, and *arn*DG206C while the second set that can be used to rule in amikacin susceptibility included *fao*AT385A, *nuo*GA890T, *nuo*GA574T, *lpt*AT55A, *lpt*AR62S, *pst*BR87C, *gid*BE126G, *gid*B Q28K, *amg*S E108Q, and *rpl*YQ41L.

Although high specificity is considered of primary importance in ruling in the diagnosis of resistance, markers showing high sensitivity and high NPV towards the phenotype of interest can be applied using the rule-out algorithm. The presence of these markers cannot confirm the phenotype or behavior of interest, but its absence practically rules out the same conclusion. This can show higher predictive performance when high sensitivity and high NPV are combined with low specificity and low LR+ in addition to high LR, high DOR and high accuracy. In the current study set, a group of efflux-pump related genes including *arm*R, *nal*C, *nal*D, *mex*R, *mex*Z, and *amp*R can be used to rule out amikacin susceptibility and thus to predict resistance and thus can be used as a first- step screening markers.

The most important predictors identified from the output of the predictive model have been functionally analyzed and results from the analysis can confirm their importance and potential effect on cell resistance. *nal*DSer32Asn has been identified as a deleterious mutant and may affect its repressor function leading to increased expression of MexAB-OprM and consequently amikacin resistance. Docking results have shown that *nal*D Ser32Asn mutant has variable binding interactions with amikacin in different tested models with some poses showing decreased amikacin binding affinity (Fig 1), reflecting a possible role of amikacin in the regulation of its own efflux by interacting with *nal*D efflux regulator, however, the exact molecular consequence of such binding needs to be further studied.

In the current analysis, both variants *fus*A1Y552C and *fus*A1D588G showed link to amikacin resistance. Mutations in the gene that encodes for elongation factor G, *fus*A, have been previously linked to fusidic acid resistance in *Salmonella Typhimurium*. Some mutations are thought to have pleiotropic effects on gene expression, which may lead to fitness differences in different environments [43]. Earle *et al*., (2016) have also reported a variety of *fus*A mutations in relation to *Staphylococcus aureus* resistance to fusidic acid [44].

Exploring the secondary structures of the gene showed that the amino acid substitution *fus*A1 Y552C occurred just at the position of the bend between the previous small alpha-helix and the subsequent long beta-strand [45] while *fus*A1D588G occurred just 2 nucleotides preceding the end of its containing beta strand. Both variants were predicted as deleterious at—2.5 cutoff with high PROVEAN scores (-6.198 and -7.256 for *fus*A1D588G and *fus*A1Y552C, respectively). Both variants were also predicted to cause large decrease in stability using I-Mutant. It appears that both *fus*A1 mutants exhibit less binding affinity to amikacin when compared to non-substituted *fus*A1 protein (Figs 2 and 3) with *fus*A1D588G showing lower binding affinities. This may explain the role of these variants in conferring amikacin resistance by interfering with amikacin binding to elongation factor G and consequently affecting its role in inhibiting protein translation. These observations highlight the important functional effect of these variants, which support their potential importance to be used as diagnostic markers. *fus*A1Y552C, has been previously reported in the literature [46, 47] and has been identified in the current analysis in relation to amikacin resistance. These observations point to the importance of *fus*A1 gene, which produces elongation factor G (EF-G1A) that is considered a key component of the translational machinery that can modify aminoglycoside susceptibility. In support of that are the previous observations of *fus*A mutations induced by the *in vitro* exposure of *Ps*. *aeruginosa* to increasing concentrations of tobramycin [48].

The Arn (PA3552-PA3559) LPS modification genes have also been linked to aminoglycosides resistance because the expression of the *arn*BCADTEF operon is recognized as a contributing factor that decreases the interaction and uptake of polycationic antibiotics through bacterial membranes [49]. Results of the current analysis support that because the two variants identified i.e., *arn*AA170T, *arn*DG206C, show higher predictive values towards amikacin resistance and were also predicted as deleterious using PROVEAN. *arn*A A170T was predicted as deleterious at -1.3 cutoff (PROVEAN score = -2.065) with predicted decrease in stability using I-Mutant while *arn*D G206C was predicted as deleterious at -2.5 cutoff (PROVEAN score = -8.374) with predicted large decrease in stability using I-Mutant. These observations may also indicate the important functional roles associated with these variants, which add to their diagnostic importance.

An important finding is the observation of a group of molecular markers showing high specificity and high positive predictive values to amikacin susceptibility. These can be regarded as molecular susceptibility markers to rule in the diagnosis of amikacin susceptibility. These include *fao*AT385A, *nuo*GA890T, *nuo*GA574T, *lpt*AT55A, and *lpt*AR62S. Screening transposon insertion mutant library has previously shown that disruption of *fao*AB and *lpt*A is associated with increased aminoglycosides susceptibility (Krahn *et al*., 2012), and these findings support findings from the current analysis. *fao*AB encodes a multienzyme complex that is involved in degradative fatty acid [FA]-oxidation, and *lpt*A encodes a lysophosphatidic acid acyltransferase (LPA), responsible for adding the second FA to glycerol-3 phosphate in the synthesis of phospholipids (PLs) [50]. Both *fao*AT385A and *lpt*AR62S were predicted as deleterious using PROVEAN. Also, each of *fao*AT385A, *lpt*AT55A, and *lpt*AR62S were also predicted to result in large decrease in protein stability, so it is proposed that these variants are good candidates to be used as amikacin susceptibility markers. This can be supported by the frequent observation of these variants in amikacin susceptible isolates, their higher predictive values towards susceptibility, and their probable functional effect in relation to amikacin binding and uptake across the outer LPS membrane of *Ps*. *aeruginosa*. Docking revealed that the *fao*AT385A variant shows decreased binding affinities to amikacin with values ranging between -5.5 and -6.2 when compared to binding affinities of the non-substituted protein which ranged between -7.2 and -7.9 (Fig 4). On the other hand, docking of *lpt*A protein (2R1A) with amikacin revealed increased affinities of the *lpt*AT55A variant with values ranging between -6 to -6.7 when compared to non-mutant, which showed binding affinities ranging between -5.2 to 6 (Fig 5). The molecular consequence of such altered binding needs to be investigated due to the complexity of steps involved in uptake and regulation of intrinsic aminoglycoside resistome, including different uptake and translational steps.

*nuo*G operon codes for proton-translocating type I NADH oxidoreductase which is an enzymatic complex that significantly contributes to the proton electrochemical gradient. Inactivation of NADH dehydrogenase has been shown to impair membrane energetics and thereby the uptake of aminoglycosides [51]. El’Garch *et al*., (2007) has previously shown that combined simultaneous mutations in *gal*U, *nuo*G, *mex*Z, and *rpl*Y can increase survival rates in *Ps*. *aeruginosa* treated with tobramycin up to 16-fold while single gene mutation has a much lower effect [51]. A recent study investigating resistance in experimentally evolved *Ps*. *aeruginosa* has identified a total of 24 mutated genes in relation to aminoglycoside resistance with ten mutants in genes directly involved in oxidative phosphorylation and proton motive force, including *nuo*G mutants [52]. Some of the identified mutants have also showed clinical relevance when re-tested. The same study has also identified *fus*A1, *mex*R, *nal*D, and *amg*S mutants as related to tobramycin resistance. In support of that are similar findings from transcriptional profile analysis, which identified the *nuo*G among other genes encoding NADH dehydrogenases and that were downregulated in adaptation to *Ps*. *aeruginosa* chronically infected lung [53]. *nuo*G mutants leading to gene disruption may also affect the survival of the organism being essential for a functional respiratory complex Ι.

On the other hand, inactivation of *pst*B has previously been linked to aminoglycoside susceptibility, especially when combined with inactivation of other genes, including those associated with lipid biosynthesis or metabolism (*lpt*A, *fao*A) or other two component regulators (amgRS) (Krahn *et al*., 2012). This study supports that by identifying *pst*BR87C variant in relation to amikacin susceptibility, which probably causes inactivation of *pst*B (PROVEAN score = - 6.874), being predicted as deleterious. It was also predicted to cause a large decrease in protein stability using I-Mutant. *Pst*B is a phosphate uptake regulatory protein that has shown to be upregulated among many other genes inducing cellular cytotoxicity under adverse conditions through phosphate acquisition [54]. These transcriptional changes showing association with cellular cytotoxicity may explain the role of the observed *pst*B variant from the current analysis. Giving additional support, other mutations in *pts*B have been previously linked to low-level tobramycin resistance [55].

*gid*B (glucose-inhibited division gene) is found among the gene cassettes harboring the OriC regions in some bacteria, including *Ps*. *aeruginosa* [56]. *gid*B is known to be involved in posttranslational modification methylation of 16S RNA. *gid*B mutant has also shown a compromised overall bacterial fitness in *Salmonella* [57]. This may reflect the physiologic cost of methylation deficiency. The effect of *gid*B mutations on antimicrobial susceptibility is thought to occur through mechanisms involving post-transcriptional modification, which explains its relation to aminoglycoside resistance. *gid*B is considered highly conserved in both Gram-positive and Gram-negative bacteria with *gid*B protein known among the proteins involved in cell cycle control of DNA replication. Consequently, its disruption may lead to inhibition of cell division which may also explain the association observed with amikacin susceptibility from the current analysis. Two deleterious amino acid substitutions in *gid*B, including E126G and Q28K showed association with amikacin susceptibility phenotype.

All the sequences identified from the current study set with the variant *gid*BE126G were amikacin susceptible. The position of this variant when compared to the tertiary structure of *gid*B methyltransferase from *Bacillus Subtilis* showed to occur at the position of the bend between the end of the third beta strand and the following alpha helix which may give an interpretation to its possible related functional role. Similar to *gid*BE126G, all the sequences with the variant *gid*B Q28K which occurs at the end of the second alpha helix [58] were amikacin susceptible. In addition to being predicted as deleterious causing large decrease in protein stability, it appears that *gid*B E126G variant leads to unfavorable states of interaction with amikacin as seen in multiple poses when compared to the non-mutant protein (Fig 6). This may indicate a decreased binding affinity of amikacin to that protein in its mutated form and probably affect its methyltransferase activity. However, such an interaction between amikacin, *gid*B, and 16S ribosomal RNA needs to be further studied.

An important point to consider when studying and interpreting the effects of different mutations, in general, is that the dynamics of mutations interactions should be overall considered. It has been shown that the buildup of resistance cannot be attributable only to DNA mutations but may also develop as a result of interactions between mutations and cellular adaptation [48]. In addition, the fitness cost of an observed mutation can lead to cross-resistance or collateral sensitivity, and this also needs to be considered. This has been previously observed with *Ps*. *aeruginosa* [59]. It is also important to consider that only 26.9% of the variance in amikacin MIC could be explained when variants were included as predictors for a better model. This finding may indicate that important elements contributing to determining amikacin resistance may have not been discovered or studied yet. The interaction between different elements may contribute to resistance or the effect of expression level could play an important role in explaining the variability in MIC.

## Conclusion

The identified sets of markers can usefully predict amikacin susceptibility phenotypes and can be used in a complementary way to guide prescription. Absence of the efflux-pump genes *arm*R, *nal*C, *nal*D, *mex*R, *mex*Z, and *amp*R as well as identifying any of the variants *nal*DSer32Asn, *fus*A1 Y552C, *fus*A1D588G, *arn*AA170T, and *arn*DG206C show high potential to guide against amikacin prescription while identifying any of the variants *fao*AT385A, *nuo*GA890T, *nuo*G A574T, *lpt*AT55A, *lpt*AR62S, *pst*BR87C, *gid*BE126G, *gid*BQ28K, *amg*SE108Q, and *rpl*YQ41L would predict amikacin susceptibility and thus recommend for its prescription. Further epidemiologic evidence and validation studies can be carried out to generalize these findings. Using such useful predictor molecular diagnostic markers would offer better informed and directed antibiotic prescription and guard against treatment failure. Such an approach would preserve available antibiotics and consequently make current treatment options viable for longer.

Aminoglycoside resistance predictors are not among the targets currently included in available diagnostic panels. It is important to highlight that currently available diagnostic panels only use acquired resistance elements as resistance predictors and do not consider chromosomal variants in most cases. Furthermore, individual agents from the same antimicrobial class are not individually addressed. On the other hand, resistance panels designed for research purposes include a large number of targets which are nonspecific or diagnostic for organism-agent combination. The suggested set of predictors can act as useful diagnostic panels to be used in a complementary way for inclusion into different sequence-based diagnostic platforms or for integration into other sequence-based clinical diagnostics workflows.

## Supporting information

S1 Table List of genome sequences included in the study with their corresponding amikacin MIC. (PDF)

S2 Table Genes and gene variants extracted from the literature. (PDF)
