# Supplementary material for: The predictive potential of different molecular markers linked to amikacin susceptibility phenotypes in Pseudomonas aeruginosa
Source: PLoS One. 2022 Apr 25;17(4):e0267396. doi: 10.1371/journal.pone.0267396 (PMC9037933; doi:10.1371/journal.pone.0267396)
Supplement: S1 Table — (PDF) [file pone.0267396.s001.pdf]

Supplementary Table S1: List of genome sequences included in the study with their corresponding amikacin MIC

Genome ID	Strain	AK MIC	BioSample Accession	Genome Length	GC Content
287.1000	AZPAE14933	32	SAMN03105631	7092428	65.74
287.1001	AZPAE14932	2	SAMN03105630	6638040	66.21
287.1002	AZPAE14931	8	SAMN03105629	6359186	66.43
287.1003	AZPAE14930	1	SAMN03105628	6302622	66.48
287.1004	AZPAE14929	128	SAMN03105627	6750744	66.09
287.1005	AZPAE14928	2	SAMN03105626	6257878	66.55
287.1006	AZPAE14927	1	SAMN03105625	6885375	65.93
287.1007	AZPAE14926	4	SAMN03105624	6886320	65.94
287.1008	AZPAE14925	2	SAMN03105623	6462755	66.29
287.1009	AZPAE14924	4	SAMN03105622	7042302	65.8
287.1010	AZPAE14923	128	SAMN03105621	6724711	66.11
287.1011	AZPAE14922	128	SAMN03105620	6839077	65.98
287.1012	AZPAE14921	2	SAMN03105619	6815406	65.99
287.1013	AZPAE14920	2	SAMN03105618	6882761	66.06
287.1014	AZPAE14919	1	SAMN03105617	6284486	66.28
287.1015	AZPAE14918	2	SAMN03105616	6318316	66.47
287.1016	AZPAE14917	8	SAMN03105615	6308307	66.52
287.1017	AZPAE14916	64	SAMN03105614	6242056	66.5
287.1018	AZPAE14915	16	SAMN03105613	6880572	65.9
287.1019	AZPAE14914	4	SAMN03105612	6858678	65.91
287.1020	AZPAE14913	2	SAMN03105611	6454858	66.27
287.1021	AZPAE14912	32	SAMN03105610	6835920	66.08
287.1022	AZPAE14911	2	SAMN03105609	6808509	65.93
287.1023	AZPAE14910	4	SAMN03105608	6253654	66.5
287.1024	AZPAE14909	4	SAMN03105607	7056926	65.94
287.1025	AZPAE14908	4	SAMN03105606	6815570	66.14
287.1026	AZPAE14907	2	SAMN03105605	6392112	66.38
287.1027	AZPAE14906	0.25	SAMN03105604	6690011	66.19
287.1028	AZPAE14905	1	SAMN03105603	6692731	66.2
287.1029	AZPAE14904	2	SAMN03105602	6937853	65.87
287.1030	AZPAE14903	1	SAMN03105601	6846030	65.86
287.1031	AZPAE14902	32	SAMN03105600	6886932	66.05
287.1032	AZPAE14901	2	SAMN03105599	6881448	66.13
287.1033	AZPAE14900	8	SAMN03105598	6851898	65.79
287.1034	AZPAE14899	2	SAMN03105597	6470623	66.16
287.1035	AZPAE14898	2	SAMN03105596	6577690	66.12

287.1036	AZPAE14897	2	SAMN03105595	6448155	66.24
287.1037	AZPAE14895	2	SAMN03105594	6330823	66.42
287.1038	AZPAE14894	0.5	SAMN03105593	7003752	65.96
287.1039	AZPAE14893	2	SAMN03105592	6769601	65.96
287.1040	AZPAE14892	4	SAMN03105591	6609151	66.05
287.1041	AZPAE14891	2	SAMN03105590	6447447	66.38
287.1042	AZPAE14890	4	SAMN03105589	7016044	65.97
287.1043	AZPAE14889	2	SAMN03105588	6479466	66.26
287.1044	AZPAE14888	2	SAMN03105587	6680559	66.15
287.1045	AZPAE14887	32	SAMN03105586	6903570	66.01
287.1046	AZPAE14886	4	SAMN03105585	7057811	65.77
287.1047	AZPAE14885	4	SAMN03105584	6406036	66.22
287.1048	AZPAE14884	4	SAMN03105583	6434371	66.45
287.1049	AZPAE14883	2	SAMN03105582	6309393	66.42
287.1050	AZPAE14882	4	SAMN03105581	6455645	66.42
287.1051	AZPAE14881	4	SAMN03105580	6703242	66.06
287.1052	AZPAE14880	4	SAMN03105579	6864526	66.11
287.1053	AZPAE14879	16	SAMN03105578	6226729	66.54
287.1054	AZPAE14878	0.25	SAMN03105577	6953921	65.92
287.1055	AZPAE14877	8	SAMN03105576	6300065	66.37
287.1056	AZPAE14876	2	SAMN03105575	6386042	66.4
287.1057	AZPAE14875	1	SAMN03105574	6980738	65.87
287.1058	AZPAE14874	8	SAMN03105573	6850351	66.05
287.1059	AZPAE14873	2	SAMN03105572	6259215	66.47
287.1060	AZPAE14872	8	SAMN03105571	7250921	65.47
287.1061	AZPAE14871	4	SAMN03105570	7054233	65.55
287.1062	AZPAE14870	128	SAMN03105569	6899104	66.03
287.1063	AZPAE14869	4	SAMN03105568	6796086	66.04
287.1064	AZPAE14868	2	SAMN03105567	6390696	66.38
287.1065	AZPAE14867	0.5	SAMN03105566	6785052	65.93
287.1066	AZPAE14866	2	SAMN03105565	6452948	66.3
287.1067	AZPAE14865	128	SAMN03105564	6894960	65.79
287.1068	AZPAE14864	2	SAMN03105563	6160523	66.57
287.1069	AZPAE14863	2	SAMN03105562	7070053	65.55
287.1070	AZPAE14862	8	SAMN03105561	7067572	65.64
287.1071	AZPAE14861	4	SAMN03105560	6713012	66.14
287.1072	AZPAE14860	0.5	SAMN03105559	6768731	66.15
287.1073	AZPAE14859	2	SAMN03105558	6527863	66.22
287.1074	AZPAE14858	2	SAMN03105557	6660551	65.95

287.1075	AZPAE14857	2	SAMN03105556	6869309	66.04
287.1076	AZPAE14856	4	SAMN03105555	6335571	66.42
287.1077	AZPAE14855	2	SAMN03105554	6342011	66.15
287.1078	AZPAE14853	128	SAMN03105553	6829167	66.09
287.1079	AZPAE14852	64	SAMN03105552	6860603	65.91
287.1080	AZPAE14851	2	SAMN03105551	6617149	66.24
287.1081	AZPAE14850	1	SAMN03105550	6729517	66.06
287.1082	AZPAE14848	0.5	SAMN03105549	6252044	66.43
287.1083	AZPAE14847	2	SAMN03105548	6345563	66.43
287.1084	AZPAE14846	8	SAMN03105547	6819636	65.85
287.1085	AZPAE14845	1	SAMN03105546	6621008	66
287.1086	AZPAE14844	2	SAMN03105545	6732772	66.12
287.1087	AZPAE14843	4	SAMN03105544	6737478	65.98
287.1088	AZPAE14842	4	SAMN03105543	6889407	65.93
287.1089	AZPAE14841	1	SAMN03105542	6845205	66.01
287.1090	AZPAE14840	32	SAMN03105541	7049244	65.61
287.1091	AZPAE14839	2	SAMN03105540	6615080	66.18
287.1092	AZPAE14838	16	SAMN03105539	7113432	65.42
287.1093	AZPAE14837	2	SAMN03105538	6449289	66.22
287.1095	AZPAE14835	2	SAMN03105536	6500090	66.12
287.1096	AZPAE14834	32	SAMN03105535	6792091	65.97
287.1097	AZPAE14833	64	SAMN03105534	6876687	65.96
287.1098	AZPAE14832	1	SAMN03105533	6543022	66.02
287.1099	AZPAE14831	8	SAMN03105532	7184792	65.56
287.1100	AZPAE14830	32	SAMN03105531	6816534	66
287.1101	AZPAE14829	0.25	SAMN03105530	6995537	66.05
287.1102	AZPAE14828	4	SAMN03105529	6872882	66.04
287.1103	AZPAE14827	4	SAMN03105528	7354112	65.59
287.1104	AZPAE14826	4	SAMN03105527	7108825	65.87
287.1105	AZPAE14825	8	SAMN03105526	6426191	66.4
287.1106	AZPAE14824	2	SAMN03105525	7034546	65.84
287.1107	AZPAE14823	2	SAMN03105524	6849147	66.04
287.1108	AZPAE14822	128	SAMN03105523	6668034	66.1
287.1109	AZPAE14821	128	SAMN03105522	6811510	66.04
287.1110	AZPAE14820	2	SAMN03105521	6364162	66.41
287.1111	AZPAE14819	128	SAMN03105520	6660543	66.16
287.1112	AZPAE14818	2	SAMN03105519	6303594	66.39
287.1113	AZPAE14817	2	SAMN03105518	6295916	66.49
287.1114	AZPAE14816	4	SAMN03105517	6738242	66.04

287.1115	AZPAE14815	1	SAMN03105516	7030097	65.89
287.1116	AZPAE14814	4	SAMN03105515	6843476	65.98
287.1117	AZPAE14813	1	SAMN03105514	6470062	66.2
287.1118	AZPAE14812	0.5	SAMN03105513	7093737	65.99
287.1119	AZPAE14811	128	SAMN03105512	6981966	65.94
287.1120	AZPAE14810	2	SAMN03105511	6316519	66.4
287.1121	AZPAE14809	4	SAMN03105510	6210274	66.51
287.1122	AZPAE14732	8	SAMN03105509	6793558	66.06
287.1123	AZPAE14731	32	SAMN03105508	6231966	66.5
287.1124	AZPAE14730	32	SAMN03105507	6865760	66.02
287.1125	AZPAE14729	128	SAMN03105506	7015575	65.78
287.1126	AZPAE14728	8	SAMN03105505	7356424	65.59
287.1127	AZPAE14727	4	SAMN03105504	7362494	65.57
287.1128	AZPAE14726	8	SAMN03105503	6885273	65.84
287.1129	AZPAE14725	16	SAMN03105502	6802459	66.14
287.1130	AZPAE14724	4	SAMN03105501	7091089	65.79
287.1131	AZPAE14723	16	SAMN03105500	6639036	66.13
287.1132	AZPAE14722	2	SAMN03105499	6781659	66.07
287.1133	AZPAE14721	8	SAMN03105498	6867543	66.01
287.1134	AZPAE14720	64	SAMN03105497	6813733	66.02
287.1135	AZPAE14719	4	SAMN03105496	6855346	66
287.1136	AZPAE14718	64	SAMN03105495	6910404	65.98
287.1137	AZPAE14717	4	SAMN03105494	7039359	65.87
287.1138	AZPAE14716	128	SAMN03105493	7038646	65.93
287.1139	AZPAE14715	16	SAMN03105492	7119339	65.79
287.1140	AZPAE14714	128	SAMN03105491	7022276	65.78
287.1141	AZPAE14713	128	SAMN03105490	7238306	65.6
287.1142	AZPAE14712	64	SAMN03105489	7010520	65.95
287.1143	AZPAE14711	16	SAMN03105488	7121620	65.79
287.1144	AZPAE14710	0.25	SAMN03105487	6748304	66.14
287.1145	AZPAE14708	8	SAMN03105486	6707536	66.05
287.1146	AZPAE14707	128	SAMN03105485	7081209	65.78
287.1147	AZPAE14706	128	SAMN03105484	6921381	66.06
287.1148	AZPAE14705	16	SAMN03105483	6888268	65.97
287.1149	AZPAE14704	2	SAMN03105482	6282527	66.51
287.1150	AZPAE14703	16	SAMN03105481	6892377	65.93
287.1151	AZPAE14702	8	SAMN03105480	7131510	65.84
287.1152	AZPAE14701	1	SAMN03105479	6990663	65.93
287.1153	AZPAE14700	32	SAMN03105478	6754658	66.09

287.1154	AZPAE14699	1	SAMN03105477	5743255	65.28
287.1155	AZPAE14698	1	SAMN03105476	6467367	65.44
287.1156	AZPAE14697	2	SAMN03105475	6531914	66.18
287.1157	AZPAE14695	2	SAMN03105474	6757024	66.06
287.1158	AZPAE14694	32	SAMN03105473	6964286	65.86
287.1159	AZPAE14693	4	SAMN03105472	6380431	66.44
287.1160	AZPAE14692	64	SAMN03105471	7011166	65.9
287.1161	AZPAE14691	4	SAMN03105470	6301926	66.57
287.1162	AZPAE14690	16	SAMN03105469	6730564	66.1
287.1163	AZPAE14689	128	SAMN03105468	7252889	65.55
287.1164	AZPAE14688	128	SAMN03105467	7007711	66.04
287.1165	AZPAE14687	128	SAMN03105466	6995963	65.89
287.1166	AZPAE14570	2	SAMN03105465	7094694	65.94
287.1167	AZPAE14566	2	SAMN03105464	6870259	65.99
287.1168	AZPAE14557	4	SAMN03105463	6772788	66.1
287.1169	AZPAE14554	8	SAMN03105462	7101679	65.75
287.1170	AZPAE14550	4	SAMN03105461	6317165	66.47
287.1171	AZPAE14538	8	SAMN03105460	6267671	66.54
287.1172	AZPAE14535	2	SAMN03105459	6944986	65.88
287.1173	AZPAE14533	1	SAMN03105458	6661879	66.27
287.1174	AZPAE14526	4	SAMN03105457	6289098	66.55
287.1175	AZPAE14509	2	SAMN03105456	6519283	66.29
287.1176	AZPAE14505	4	SAMN03105455	6823044	66.04
287.1177	AZPAE14499	0.25	SAMN03105454	6698732	66.12
287.1178	AZPAE14463	128	SAMN03105453	7034157	65.85
287.1179	AZPAE14453	128	SAMN03105452	7052856	65.76
287.1180	AZPAE14443	1	SAMN03105451	6268752	66.59
287.1181	AZPAE14442	128	SAMN03105450	7007955	65.97
287.1182	AZPAE14441	4	SAMN03105449	6810460	65.93
287.1183	AZPAE14437	4	SAMN03105448	6714753	66.04
287.1184	AZPAE14422	4	SAMN03105447	6732223	66.05
287.1185	AZPAE14415	8	SAMN03105446	6982687	65.83
287.1186	AZPAE14410	2	SAMN03105445	6295059	66.5
287.1187	AZPAE14404	4	SAMN03105444	6575587	66.3
287.1188	AZPAE14403	4	SAMN03105443	6907387	66.08
287.1189	AZPAE14402	4	SAMN03105442	6466306	66.33
287.1190	AZPAE14398	2	SAMN03105441	6693786	66.09
287.1191	AZPAE14395	0.5	SAMN03105440	6325978	66.49
287.1192	AZPAE14394	4	SAMN03105439	6977785	66.01

287.1193	AZPAE14393	2	SAMN03105438	6723039	66.23
287.1194	AZPAE14390	2	SAMN03105437	6884000	65.97
287.1195	AZPAE14381	64	SAMN03105436	6791911	65.83
287.1196	AZPAE14379	2	SAMN03105435	6253630	66.51
287.1197	AZPAE14373	2	SAMN03105434	6337010	66.43
287.1198	AZPAE14372	8	SAMN03105433	6794294	66.13
287.1199	AZPAE14359	4	SAMN03105432	6642619	66.29
287.1200	AZPAE14353	4	SAMN03105431	6835254	66.01
287.1201	AZPAE14352	0.5	SAMN03105430	6561378	66
287.1202	AZPAE13880	64	SAMN03105429	6855952	66
287.1203	AZPAE13879	2	SAMN03105428	6948193	65.92
287.1204	AZPAE13877	64	SAMN03105427	6788523	66.1
287.1205	AZPAE13876	0.12	SAMN03105426	6795623	65.85
287.1206	AZPAE13872	32	SAMN03105425	6958295	65.9
287.1207	AZPAE13866	2	SAMN03105424	7258328	65.73
287.1208	AZPAE13864	32	SAMN03105423	6909435	65.95
287.1209	AZPAE13860	128	SAMN03105422	6888735	65.91
287.1210	AZPAE13858	32	SAMN03105421	7017824	66.02
287.1211	AZPAE13856	64	SAMN03105420	7373661	65.7
287.1212	AZPAE13853	32	SAMN03105419	7040099	65.99
287.1213	AZPAE13850	64	SAMN03105418	5501656	65.73
287.1214	AZPAE13848	16.001	SAMN03105417	6958460	66.07
287.1215	AZPAE13757	128	SAMN03105416	6469470	66.38
287.1216	AZPAE13756	128	SAMN03105415	7335469	65.54
287.1217	AZPAE12423	64	SAMN03105414	6535360	66.21
287.1218	AZPAE12422	32	SAMN03105413	6331130	66.45
287.1219	AZPAE12421	32	SAMN03105412	6243887	66.4
287.1220	AZPAE12420	16	SAMN03105411	6283659	66.37
287.1221	AZPAE12419	16	SAMN03105410	6290772	66.46
287.1222	AZPAE12418	32	SAMN03105409	6737495	66.21
287.1223	AZPAE12417	16	SAMN03105408	6360200	66.3
287.1224	AZPAE12416	64	SAMN03105407	6527924	66.15
287.1225	AZPAE12415	32	SAMN03105406	6740389	66.26
287.1226	AZPAE12414	2	SAMN03105405	6736819	66.28
287.1227	AZPAE12413	32	SAMN03105404	6742361	66.26
287.1228	AZPAE12412	4	SAMN03105403	6445104	66.39
287.1229	AZPAE12411	2	SAMN03105402	6341911	66.42
287.1230	AZPAE12410	4	SAMN03105401	6457280	66.37
287.1231	AZPAE12409	8	SAMN03105400	6758579	65.98

287.1232	AZPAE12156	32	SAMN03105399	6571733	66.05
287.1233	AZPAE12155	8	SAMN03105398	6732273	66.12
287.1234	AZPAE12154	64	SAMN03105397	6299347	66.38
287.1235	AZPAE12153	128	SAMN03105396	6193644	66.55
287.1237	AZPAE12151	128	SAMN03105394	6332026	66.47
287.1238	AZPAE12150	8	SAMN03105393	6594453	65.98
287.1239	AZPAE12149	128	SAMN03105392	6355203	66.36
287.1240	AZPAE12148	32	SAMN03105391	6650984	66.28
287.1241	AZPAE12147	64	SAMN03105390	6522044	66.25
287.1242	AZPAE12146	8	SAMN03105389	6313502	66.44
287.1243	AZPAE12145	128	SAMN03105388	6452923	66.4
287.1245	AZPAE12143	16	SAMN03105386	6473536	66.34
287.1246	AZPAE12142	128	SAMN03105385	6323807	66.42
287.1247	AZPAE12140	16	SAMN03105384	6250380	66.55
287.1248	AZPAE12138	128	SAMN03105383	6576673	66.14
287.1249	AZPAE12137	64	SAMN03105382	6304247	66.52
287.1250	AZPAE12136	4	SAMN03105381	6303512	66.43
287.1301	AZPAE12135	8	SAMN03105380	6826162	66.07
287.1477	MRSN 20176	64	SAMN03316892	6730817	65.93
287.1482	MRSN18971	8	SAMN02673308	6391538	66.45
287.2972	AR_0103	32	SAMN04014944	6900175	65.99
287.2973	AR_0092	64	SAMN04014933	6963676	66.02
287.2975	AR_0100	64	SAMN04014941	6929574	65.98
287.2976	AR_0105	8	SAMN04014946	6425652	66.22
287.2977	AR_0054	2	SAMN04014895	7267705	65.81
287.2978	AR_0064	1	SAMN04014905	6827144	66.05
287.2979	AR_0094	32	SAMN04014935	6882581	66.05
287.2980	AR_0108	64	SAMN04014949	6971125	65.98
287.5685	AR_0360	4	SAMN07291503	6463575	66.44145
287.5686	AR_0354	8	SAMN07291497	6747010	66.09722
287.5687	AR_0353	64	SAMN07291496	7282236	65.704834
287.5688	AR_0230	64	SAMN04901620	7086054	65.87829
287.5689	AR_0356	2	SAMN07291499	7247865	65.59437
287.5690	AR_0357	64	SAMN07291500	7162784	65.79707
287.5701	AR_0355	8	SAMN07291498	7172470	65.84778
287.5702	AR_0359	4	SAMN07291502	6940345	65.91524
287.5703	AR_0358	4	SAMN07291501	7284124	65.71017
287.5704	AR_0351	64	SAMN07291494	6611054	66.24956
287.5746	AR_0443	16	SAMN07291536	6776714	65.83632

287.5747	AR_0440	16	SAMN07291533	7167215	65.84894
287.5748	AR439	64	SAMN07291532	7578039	65.418785
287.5749	AR442	4	SAMN07291535	7267567	65.752335
287.5750	AR445	64	SAMN07291538	7125975	65.78442
287.5751	AR441	1	SAMN07291534	7245771	65.59486
287.5752	AR444	64	SAMN07291537	6853499	66.05279
287.5778	AR_0095	1	SAMN04014936	6822666	66.10762
287.5955	AR_0446	4	SAMN07291539	6475581	66.33031
287.5956	CCUG 70744	16	SAMN07602569	6859232	66.04034
287.5959	AR_0110	64	SAMN04014951	6799785	66.01369
287.5969	AR_0447	8	SAMN07291540	7178792	65.86686
287.5971	AR_460	64	SAMN07291553	6303875	66.55481
287.5972	AR_455	1	SAMN07291548	6540996	65.8842
287.5973	AR_458	16	SAMN07291551	6685102	66.23354
287.6326	AR_0459	8	SAMN07291552	6752712	66.24611
287.6327	AR_0457	2	SAMN07291550	7381251	65.63855
287.6328	AR_0456	8	SAMN07291549	7079305	65.857216
287.6329	AR_0449	8	SAMN07291542	6792240	65.96245
287.6330	AR_0241	32	SAMN04901631	7226502	65.64898
287.6331	AR_0352	2	SAMN07291495	6418505	66.43134
287.6492	AR_0111	64	SAMN04014952	7075653	65.830505
287.7771	MRSN8914	32	SAMN09788319	7356800	65.33966
287.7772	MRSN8915	8	SAMN09788318	7064713	65.680504
287.7773	MRSN994	8	SAMN09788315	6734108	66.12464
287.7774	MRSN8139	8	SAMN09788322	6575059	66.36821
287.7775	MRSN7014	16	SAMN09788325	6420453	66.4322
287.7776	MRSN8912	8	SAMN09788320	6986301	65.71874
287.7777	MRSN8136	8	SAMN09788323	6907900	65.713806
287.7778	MRSN6695	8	SAMN09788327	6235816	66.576324
287.7779	MRSN6241	8	SAMN09788329	7161217	65.6252
287.7780	MRSN6678	8	SAMN09788328	6782774	66.11963
287.7781	MRSN6220	32	SAMN09788330	6910198	66.00948
287.7782	MRSN5524	8	SAMN09788332	6725073	65.99564
287.7783	MRSN5539	8	SAMN09788331	6769662	66.07058
287.7784	MRSN552	8	SAMN09788333	6444919	66.35499
287.7785	MRSN5519	32	SAMN09788334	6751302	66.13514
287.7786	MRSN4841	8	SAMN09788337	6475204	66.25277
287.7787	MRSN5498	8	SAMN09788336	6821230	66.00017
287.7788	MRSN443463	8	SAMN09788338	6393241	66.4404

287.7789	MRSN315	8	SAMN09788352	6696919	66.20516
287.7790	MRSN19711	8	SAMN09788366	6265327	66.576096
287.7791	MRSN20190	32	SAMN09788365	6191883	66.59317
287.7792	MRSN9873	8	SAMN09788316	7146707	65.48819
287.7793	MRSN9718	8	SAMN09788317	6861353	66.019516
287.7794	MRSN8141	8	SAMN09788321	7161718	65.6272
287.7795	MRSN8130	8	SAMN09788324	6828028	66.09563
287.7796	MRSN6739	8	SAMN09788326	6298064	66.478065
287.7797	MRSN5508	8	SAMN09788335	6439859	66.34678
287.7798	MRSN435288	16	SAMN09788340	6363888	66.22477
287.7799	MRSN321	8	SAMN09788350	6260875	66.54782
287.7800	MRSN25678	8	SAMN09788357	6509751	66.21528
287.7801	MRSN18754	8	SAMN09788375	6270751	66.541534
287.7802	MRSN390231	8	SAMN09788343	6149846	66.37745
287.7803	MRSN436311	8	SAMN09788339	6473360	66.416046
287.7804	MRSN373401	8	SAMN09788344	6985982	65.97148
287.7805	MRSN3705	8	SAMN09788345	6419743	66.576324
287.7806	MRSN29192	8	SAMN09788354	6375161	66.43941
287.7807	MRSN25762	8	SAMN09788356	6845305	66.2181
287.7808	MRSN2444	16	SAMN09788359	6843047	66.06668
287.7809	MRSN3587	8	SAMN09788364	6494873	66.36721
287.7810	MRSN2101	8	SAMN09788363	6346725	66.376564
287.7811	MRSN23861	8	SAMN09788360	7025487	65.94264
287.7812	MRSN20176	32	SAMN09788414	6717057	65.99654
287.7813	MRSN1948	8	SAMN09788367	6674435	66.19558
287.7814	MRSN1906	8	SAMN09788370	7049219	65.7835
287.7815	MRSN1938	8	SAMN09788368	7052906	66.11615
287.7816	MRSN1899	8	SAMN09788372	6573139	66.22041
287.7817	MRSN1902	8	SAMN09788371	6344431	66.457214
287.7818	MRSN18970	8	SAMN09788413	6370604	66.51189
287.7819	MRSN17849	8	SAMN09788378	6738933	66.124664
287.7820	MRSN16744	8	SAMN09788382	6776946	66.11751
287.7821	MRSN16740	8	SAMN09788383	6670940	66.08019
287.7822	MRSN1688	8	SAMN09788380	6606996	66.148384
287.7823	MRSN16383	16	SAMN09788384	6302224	66.533325
287.7824	MRSN16345	8	SAMN09788385	6333273	66.51163
287.7825	MRSN1613	8	SAMN09788388	6276349	66.510056
287.7826	MRSN1601	8	SAMN09788390	6411494	66.28397
287.7827	MRSN1583	8	SAMN09788391	6463376	66.37041

287.7828	MRSN1617	8	SAMN09788387	6780137	66.19642
287.7829	MRSN1388	8	SAMN09788396	7171428	65.862366
287.7830	MRSN15678	8	SAMN09788393	6780257	66.094574
287.7831	MRSN14981	8	SAMN09788395	6508561	66.21667
287.7832	MRSN409937	8	SAMN09788341	6487691	66.33823
287.7833	MRSN351791	8	SAMN09788348	6812488	65.93882
287.7834	MRSN346179	8	SAMN09788349	6293739	66.51709
287.7835	MRSN30858	8	SAMN09788353	6382502	66.4238
287.7836	MRSN26263	8	SAMN09788355	6430277	66.320595
287.7837	MRSN1925	8	SAMN09788369	7130564	65.83213
287.7838	MRSN25623	8	SAMN09788358	6635285	66.08652
287.7839	MRSN18562	8	SAMN09788376	6494310	66.368095
287.7840	MRSN16344	32	SAMN09788386	6318735	66.457306
287.7841	MRSN1612	8	SAMN09788389	6506983	66.235
287.7842	MRSN1739	8	SAMN09788379	7031489	65.82428
287.7843	MRSN16847	8	SAMN09788381	6594082	65.978676
287.7844	MRSN15753	8	SAMN09788392	6547438	66.18349
287.7845	MRSN1380	8	SAMN09788397	6339603	66.48981
287.7846	MRSN15566	8	SAMN09788394	6408848	66.389984
287.7847	MRSN1356	8	SAMN09788398	6555630	66.19885
287.7848	MRSN12914	16	SAMN09788401	6756810	65.92381
287.7849	MRSN12283	8	SAMN09788404	6301640	66.499626
287.7850	MRSN12365	16	SAMN09788403	6869499	65.87287
287.7851	MRSN358800	32	SAMN09788347	6501460	66.35765
287.7852	MRSN11538	8	SAMN09788407	6528585	66.261604
287.7853	MRSN11278	32	SAMN09788412	6893110	66.043076
287.7854	MRSN11281	8	SAMN09788411	6323478	66.49251
287.7855	MRSN13488	8	SAMN09788399	5912421	66.50286
287.7856	MRSN1344	8	SAMN09788400	6232278	66.494484
287.7857	MRSN12368	16	SAMN09788402	6340159	66.43633
287.7858	MRSN11285	8	SAMN09788410	6650291	66.16359
287.7859	MRSN12282	8	SAMN09788405	6876949	65.96845
287.7860	MRSN11536	8	SAMN09788408	6935556	65.91659
287.7861	MRSN11286	8	SAMN09788409	6711313	65.911575
287.7862	MRSN11976	8	SAMN09788406	6991711	65.90075
287.8027	MRSN401528	8	SAMN09788342	6486368	66.14036
287.8028	MRSN369569	32	SAMN09788346	6316238	66.518555
287.8029	MRSN2144	8	SAMN09788361	7032738	65.79075
287.8030	MRSN18855	8	SAMN09788373	6296953	66.489555

287.8031	MRSN2108	8	SAMN09788362	6394452	66.39729
287.8032	MRSN18803	8	SAMN09788374	6191380	66.5096
287.8033	MRSN317	8	SAMN09788351	6349621	66.47357
287.8034	MRSN18560	8	SAMN09788377	6268481	66.5069
287.846	AZPAE15072	8	SAMN03105769	6437786	66.3
287.847	AZPAE15071	4	SAMN03105768	7403124	65.72
287.848	AZPAE15070	4	SAMN03105767	7164330	65.83
287.849	AZPAE15069	2	SAMN03105766	6980745	65.93
287.850	AZPAE15068	2	SAMN03105765	6856096	66.14
287.851	AZPAE15067	2	SAMN03105764	6651822	66.15
287.852	AZPAE15066	4	SAMN03105763	6959180	65.82
287.853	AZPAE15065	32	SAMN03105762	6184461	66.47
287.854	AZPAE15064	4	SAMN03105761	6687065	66.19
287.855	AZPAE15063	64	SAMN03105760	6768150	66.03
287.856	AZPAE15062	2	SAMN03105759	6481258	66.31
287.857	AZPAE15061	2	SAMN03105758	6832661	65.96
287.858	AZPAE15060	4	SAMN03105757	6716256	66.11
287.859	AZPAE15059	4	SAMN03105756	6657813	66.25
287.860	AZPAE15058	1	SAMN03105755	6567883	66.22
287.861	AZPAE15057	4	SAMN03105754	6451298	66.43
287.862	AZPAE15056	4	SAMN03105753	6287511	66.49
287.863	AZPAE15055	4	SAMN03105752	6360589	66.37
287.864	AZPAE15054	2	SAMN03105751	6647129	65.96
287.865	AZPAE15053	2	SAMN03105750	6522359	66.25
287.866	AZPAE15052	2	SAMN03105749	6339616	66.38
287.867	AZPAE15051	4	SAMN03105748	6814130	66.09
287.868	AZPAE15050	1	SAMN03105747	6901290	65.78
287.869	AZPAE15049	2	SAMN03105746	6259893	66.46
287.870	AZPAE15048	2	SAMN03105745	6924072	65.73
287.871	AZPAE15047	128	SAMN03105744	6898282	66.03
287.872	AZPAE15046	16	SAMN03105743	6345609	66.45
287.873	AZPAE15045	2	SAMN03105742	6703034	66
287.874	AZPAE15044	4	SAMN03105741	6555614	66.21
287.875	AZPAE15043	4	SAMN03105740	6978967	65.98
287.876	AZPAE15042	1	SAMN03105739	6640508	66.27
287.877	AZPAE15041	2	SAMN03105738	6739630	66.02
287.878	AZPAE15040	4	SAMN03105737	6601216	66.23
287.879	AZPAE15039	0.25	SAMN03105736	6697373	66.07
287.880	AZPAE15038	4	SAMN03105735	6261442	66.49

287.881	AZPAE15037	8	SAMN03105734	7200442	65.83
287.882	AZPAE15036	1	SAMN03105733	6412424	66.31
287.883	AZPAE15035	2	SAMN03105732	6673047	66.09
287.884	AZPAE15034	2	SAMN03105731	6794620	66.14
287.885	AZPAE15033	0.25	SAMN03105730	6087226	66.4
287.886	AZPAE15032	1	SAMN03105729	6484573	66.2
287.887	AZPAE15031	2	SAMN03105728	6894718	65.98
287.888	AZPAE15030	8	SAMN03105727	6608631	66.18
287.889	AZPAE15029	128	SAMN03105726	6860916	66.03
287.890	AZPAE15028	2	SAMN03105725	6834546	65.7
287.891	AZPAE15027	2	SAMN03105724	6438293	66.36
287.892	AZPAE15026	1	SAMN03105723	6358197	66.41
287.893	AZPAE15025	2	SAMN03105722	6726954	66
287.894	AZPAE15024	2	SAMN03105721	6776478	66
287.895	AZPAE15023	2	SAMN03105720	6351355	66.4
287.896	AZPAE15022	1	SAMN03105719	6219362	66.56
287.897	AZPAE15021	64	SAMN03105718	6686424	66.05
287.899	AZPAE15019	2	SAMN03105716	6717129	66.06
287.900	AZPAE15018	2	SAMN03105715	6933997	66.06
287.901	AZPAE15017	4	SAMN03105714	6594600	66.09
287.902	AZPAE15016	4	SAMN03105713	6756824	65.84
287.903	AZPAE15015	8	SAMN03105712	6804229	66.08
287.904	AZPAE15014	2	SAMN03105711	6400436	66.42
287.905	AZPAE15013	1	SAMN03105710	6358832	66.41
287.906	AZPAE15012	4	SAMN03105709	6462525	66.32
287.907	AZPAE15011	1	SAMN03105708	6590340	65.85
287.908	AZPAE15010	2	SAMN03105707	7014236	65.9
287.909	AZPAE15009	1	SAMN03105706	6958019	65.85
287.910	AZPAE15008	1	SAMN03105705	6345734	66.18
287.911	AZPAE15007	1	SAMN03105704	6814810	65.79
287.912	AZPAE15006	4	SAMN03105703	6832413	65.95
287.913	AZPAE15005	4	SAMN03105702	6889490	66.07
287.914	AZPAE15004	2	SAMN03105701	6345076	66.39
287.915	AZPAE15003	128	SAMN03105700	6882469	66.11
287.916	AZPAE15002	64	SAMN03105699	6802969	65.79
287.917	AZPAE15001	2	SAMN03105698	6582180	66.3
287.918	AZPAE15000	16	SAMN03105697	6858205	66
287.919	AZPAE14999	1	SAMN03105696	6427887	66.3
287.920	AZPAE14998	1	SAMN03105695	6712745	66.17

287.921	AZPAE14997	1	SAMN03105694	6715214	65.98
287.922	AZPAE14996	2	SAMN03105693	6459808	66.34
287.923	AZPAE14995	2	SAMN03105692	6389451	66.32
287.924	AZPAE14994	1	SAMN03105691	6476787	66.07
287.925	AZPAE14993	2	SAMN03105690	6914857	65.97
287.926	AZPAE14992	1	SAMN03105689	6510002	66.3
287.927	AZPAE14991	2	SAMN03105688	6467607	66.31
287.928	AZPAE14990	2	SAMN03105687	6809137	66.09
287.929	AZPAE14989	2	SAMN03105686	6549370	66.19
287.930	AZPAE14988	2	SAMN03105685	6387737	66.41
287.931	AZPAE14987	2	SAMN03105684	6855155	66.02
287.932	AZPAE14986	1	SAMN03105683	6471510	66.35
287.933	AZPAE14985	2	SAMN03105682	6781336	66.05
287.934	AZPAE14984	128	SAMN03105681	6809111	66.07
287.951	AZPAE14983	16	SAMN03105680	7082650	65.77
287.952	AZPAE14982	2	SAMN03105679	6455689	66.31
287.953	AZPAE14981	4	SAMN03105678	6276540	66.48
287.954	AZPAE14980	4	SAMN03105677	6768315	66.16
287.955	AZPAE14979	8	SAMN03105676	6751299	66.03
287.956	AZPAE14978	4	SAMN03105675	7130480	65.74
287.957	AZPAE14977	2	SAMN03105674	6336142	66.39
287.958	AZPAE14976	4	SAMN03105673	6631621	66.06
287.959	AZPAE14975	2	SAMN03105672	6369755	66.42
287.960	AZPAE14974	0.5	SAMN03105671	7085926	65.75
287.961	AZPAE14973	1	SAMN03105670	7183565	65.78
287.962	AZPAE14972	1	SAMN03105669	6399923	66.38
287.963	AZPAE14971	0.12	SAMN03105668	6203904	66.44
287.964	AZPAE14970	4	SAMN03105667	6920457	66.05
287.965	AZPAE14969	4	SAMN03105666	6832241	66
287.966	AZPAE14968	1	SAMN03105665	6897223	66
287.967	AZPAE14967	1	SAMN03105664	6567690	66.17
287.968	AZPAE14965	1	SAMN03105663	7042523	65.91
287.969	AZPAE14964	1	SAMN03105662	7039582	65.91
287.970	AZPAE14963	1	SAMN03105661	6380431	66.44
287.971	AZPAE14962	2	SAMN03105660	6963110	65.83
287.972	AZPAE14961	1	SAMN03105659	6441305	66.34
287.973	AZPAE14960	2	SAMN03105658	6370668	66.45
287.974	AZPAE14959	64	SAMN03105657	6743682	66.15
287.975	AZPAE14958	64	SAMN03105656	7130390	65.74

287.976	AZPAE14957	2	SAMN03105655	6621214	66.11
287.977	AZPAE14956	128	SAMN03105654	7110106	65.36
287.978	AZPAE14955	1	SAMN03105653	6375587	66.42
287.979	AZPAE14954	2	SAMN03105652	6368200	66.43
287.980	AZPAE14953	2	SAMN03105651	6809063	66.15
287.981	AZPAE14952	2	SAMN03105650	6365190	66.39
287.982	AZPAE14951	64	SAMN03105649	6876017	65.85
287.983	AZPAE14950	2	SAMN03105648	6332797	66.24
287.984	AZPAE14949	32	SAMN03105647	6899528	66.05
287.985	AZPAE14948	16	SAMN03105646	6828755	65.86
287.986	AZPAE14947	16	SAMN03105645	6482138	66.33
287.987	AZPAE14946	4	SAMN03105644	6592636	66.29
287.988	AZPAE14945	4	SAMN03105643	6866953	66.05
287.989	AZPAE14944	0.12	SAMN03105642	6824257	66.11
287.990	AZPAE14943	1	SAMN03105641	6250531	66.53
287.991	AZPAE14942	1	SAMN03105640	6787557	66.05
287.992	AZPAE14941	1	SAMN03105639	6881480	65.98
287.993	AZPAE14940	4	SAMN03105638	6929735	65.97
287.994	AZPAE14939	16	SAMN03105637	6351078	66.39
287.995	AZPAE14938	4	SAMN03105636	6900154	66.12
287.996	AZPAE14937	8	SAMN03105635	6892469	65.86
287.997	AZPAE14936	0.5	SAMN03105634	6265859	66.33
287.998	AZPAE14935	4	SAMN03105633	6764634	66.22
287.999	AZPAE14934	2	SAMN03105632	6346157	66.37
