# Supplementary material for: The predictive potential of different molecular markers linked to amikacin susceptibility phenotypes in Pseudomonas aeruginosa
Source: PLoS One. 2022 Apr 25;17(4):e0267396. doi: 10.1371/journal.pone.0267396 (PMC9037933; doi:10.1371/journal.pone.0267396)
Supplement: S2 Table — (PDF) [file pone.0267396.s002.pdf]

Supplementary Table S2: Genes and gene variants extracted from the literature

*nal*B mutants<br>Mutation in *mex*R leading to overexpression in efflux pump	Arg70Trp, Leu80Pro, Arg91His, Arg83His,<br>Leu57Arg, Arg59Cys, Gly58Glu, Leu95Phe,<br>Thr130Pro, Thr69Ile, Arg21Trp<br>Leu13Met, Ala110Thr<br>Asp8Gly, Ala66His, Lys44Ser	(Adewoye *et al.*, 2002)<br>(Choudhury *et al.*, 2016)
	Arg70Trp, Thr130Pro<br>Gln94Pro, Leu57Arg	(Srikumar, Paul and Poole, 2000)
	Pro7leu, Pro143Thr, Val126Glu	(Suresh *et al.*, 2018)
	Asp8Glu, Ala66val	(Ziha-Zarifi *et al.*, 1999)
	Lys44Met, Val126Glu, Ala66Pro	(Llanes *et al.*, 2004)
	His107Pro, Ala103Thr, Gln106His,<br>Ser26Gly, Asn79Gly, Met10Arg, Ser88Cys<br>Asn53Asp, Asn53Tyr, Arg21Gly	(Higgins *et al.*, 2003)
	Val-126-Glu	(Vaez *et al.*, 2014)
*nal*C mutants	Gly71Glu, Ser46Ala, Glu153Gln,<br>Met151Thr, Ser209Arg, Asp76Glu, Leu61Pro	(Llanes *et al.*, 2004)
	Ser127Pro, Thr50Pro	(Cao, Srikumar and Poole, 2004)
*nal*D mutants	Ser32Asn<br>Change at nucloetideT410<br>Change at nucleotide G433	(Sobel *et al.*, 2005)
	Phe175Ala	(Chen *et al.*, 2016)
	Leu22Pro, Thr158Ile	(Jorth *et al.*, 2017)
	Leu153Gln	(Suresh *et al.*, 2018)
*nfx*B	Ala124Glu, Glu111Lys, Arg21His,	(Higgins *et al.*, 2003)

	Gln52His, Ala141Gly, Ser36Gly, Ala38Gly, Asp56Gly, Glu8Lys, Gln64His,Glu75Gln	
	Gly166Asp, Gly192Asp, Phe147Ser	(Purssell and Poole, 2013)
	Arg42Gly, Arg42His	(Chuanchuen *et al.*, 2001)
	Glu124Ala	(Vaez *et al.*, 2014)
	Arg82Leu	(Jalal *et al.*, 2000)
*nfx*C	*mex*S Asn249Asp, *mex*S Val104Ala, *mexS Phe253Leu, mexS Leu263Gln*	(Richardot *et al.*, 2016)
	*mex*S Glu54Gly, *mex*S Gly78Ser<br>*mex*S Ala75Val, *mex*S Thr152Ala<br>*mex*S Ala175Val, *mex*S Glu181Asp<br>*mex*S Val308Ile, *mex*S Cys269Yyr	(Llanes *et al.*, 2011)
	*mex*S Asp244Asn, *mex*S Val333Gly<br>*mex*S Ser124Arg	(Sobel, Neshat and Poole, 2005)
	*mex*T Gly258Asp, *mex*T Yyr138Asp	(Richardot *et al.*, 2016)
	*mex*T Gly257Ser, *mex*T Arg166His	(Llanes *et al.*, 2011)
*amp*R	Gly154Arg, Glu114Ala, Gly283Glu<br>Met288Arg, Ala51Thr	(Cabot *et al.*, 2012)
	Asp135Asn, Gly102Glu	(Caille *et al.*, 2014)
*gid*B	T230C, C286T, T104G, A254G	(Sirgel *et al.*, 2012)<br>(Maus, Plikaytis and Shinnick, 2005)<br>(Nessar *et al.*, 2011)<br>(Springer *et al.*, 2001)
*amg*S	Arg182Cys, Val121Gly, Asp106Asn<br>Ala28Glu, Cys28Ala	(Lau *et al.*, 2015)<br>(Lau *et al.*, 2013)<br>(Schniederjans, Koska and Häussler, 2017)
*pmr*A	Leu71Arg, Asp92Tyr	(Schniederjans, Koska and Häussler, 2017)
*pmr*B	Thr4Ala, Leu323His, Ser420Arg	(Schniederjans, Koska and Häussler, 2017)
	Gly423Cys, Leu243Gln, Ala248Val	(Moskowitz, Ernst and Miller, 2004)
fusA1 (PA4266)	Yyr552Cys, Arg371Cys, Thr456Ala	(Bolard, Plésiat and Jeannot, 2018)

	Arg680Cys, Val93Ala, Ala555Glu<br>Thr671Ala	(López-Causapé *et al.*, 2018)
*rpl*Y (PA4671)	Gly367Thr, Ala123Ser	(Islam *et al.*, 2009)<br>(Poonsuk, Tribuddharat and Chuanchuen, 2013)

References:

[1] Adewoye L, Sutherland A, Srikumar R, Poole K. The mexR repressor of the mexAB-oprM multidrug efflux operon in Pseudomonas aeruginosa: characterization of mutations compromising activity. J Bacteriol 2002;184:4308–12.

[2] Choudhury D, Ghosh A, Dhar Chanda D, Das Talukdar A, Dutta Choudhury M, Paul D, et al. Premature Termination of MexR Leads to Overexpression of MexAB-OprM Efflux Pump in Pseudomonas aeruginosa in a Tertiary Referral Hospital in India. PLoS One 2016;11:e0149156. https://doi.org/10.1371/journal.pone.0149156.

[3] Srikumar R, Paul CJ, Poole K. Influence of mutations in the mexR repressor gene on expression of the MexA-MexB-oprM multidrug efflux system of Pseudomonas aeruginosa. J Bacteriol 2000;182:1410–4.

[4] Suresh M, Nithya N, Jayasree PR, Vimal KP, Manish Kumar PR. Mutational analyses of regulatory genes, mexR, nalC, nalD and mexZ of mexAB-oprM and mexXY operons, in efflux pump hyperexpressing multidrug-resistant clinical isolates of Pseudomonas aeruginosa. World J Microbiol Biotechnol 2018;34:83. https://doi.org/10.1007/s11274-018-2465-0.

[5] Ziha-Zarifi I, Llanes C, Köhler T, Pechere JC, Plesiat P. In vivo emergence of multidrug-resistant mutants of Pseudomonas aeruginosa overexpressing the active efflux system MexA-MexB-OprM. Antimicrob Agents Chemother 1999;43:287–91.

[6] Llanes C, Hocquet D, Vogne C, Benali-Baitich D, Neuwirth C, Plésiat P. Clinical strains of Pseudomonas aeruginosa overproducing MexAB-OprM and MexXY efflux pumps simultaneously. Antimicrob Agents Chemother 2004;48:1797–802.

[7] Higgins PG, Fluit AC, Milatovic D, Verhoef J, Schmitz F-J. Mutations in GyrA, ParC, MexR and NfxB in clinical isolates of Pseudomonas aeruginosa. Int J Antimicrob Agents 2003;21:409–13.

[8] Vaez H, Faghri J, Isfahani BN, Moghim S, Yadegari S, Fazeli H, et al. Efflux pump regulatory genes mutations in multidrug resistance Pseudomonas aeruginosa isolated from wound infections in Isfahan hospitals. Adv Biomed Res 2014;3:117. https://doi.org/10.4103/2277-9175.133183.

[9] Cao L, Srikumar R, Poole K. MexAB-OprM hyperexpression in NalC-type multidrug-resistant Pseudomonas aeruginosa: identification and characterization of the nalC gene encoding a repressor of PA3720-PA3719. Mol Microbiol 2004;53:1423–36. https://doi.org/10.1111/j.1365-2958.2004.04210.x.

[10] Sobel ML, Hocquet D, Cao L, Plesiat P, Poole K. Mutations in PA3574 (nalD) lead to increased MexAB-OprM expression and multidrug resistance in laboratory and clinical isolates of Pseudomonas aeruginosa. Antimicrob Agents Chemother 2005;49:1782–6. https://doi.org/10.1128/AAC.49.5.1782-1786.2005.

[11] Chen W, Wang D, Zhou W, Sang H, Liu X, Ge Z, et al. Novobiocin binding to NalD induces the expression of the MexAB-OprM pump in Pseudomonas aeruginosa. Mol Microbiol 2016;100:749–58. https://doi.org/10.1111/mmi.13346.

[12] Jorth P, McLean K, Ratjen A, Secor PR, Bautista GE, Ravishankar S, et al. Evolved Aztreonam Resistance Is Multifactorial and Can Produce Hypervirulence in Pseudomonas aeruginosa. MBio 2017;8. https://doi.org/10.1128/mBio.00517-17.

[13] Purssell A, Poole K. Functional characterization of the NfxB repressor of the mexCD-oprJ multidrug efflux operon of Pseudomonas aeruginosa. Microbiology 2013;159:2058–73. https://doi.org/10.1099/mic.0.069286-0.

[14] Chuanchuen R, Beinlich K, Hoang TT, Becher A, Karkhoff-Schweizer RR, Schweizer HP. Cross-resistance between triclosan and antibiotics in Pseudomonas aeruginosa is mediated by multidrug efflux pumps: exposure of a susceptible mutant strain to triclosan selects nfxB mutants overexpressing MexCD-OprJ. Antimicrob Agents Chemother 2001;45:428–32. https://doi.org/10.1128/AAC.45.2.428-432.2001.

[15] Jalal S, Ciofu O, Hoiby N, Gotoh N, Wretlind B. Molecular mechanisms of fluoroquinolone resistance in Pseudomonas aeruginosa isolates from cystic fibrosis patients. Antimicrob Agents Chemother 2000;44:710–2.

[16] Richardot C, Juarez P, Jeannot K, Patry I, Plésiat P, Llanes C. Amino Acid Substitutions Account for Most MexS Alterations in Clinical nfxC Mutants of Pseudomonas aeruginosa. Antimicrob Agents Chemother 2016;60:2302–10. https://doi.org/10.1128/AAC.02622-15.

[17] Llanes C, Köhler T, Patry I, Dehecq B, van Delden C, Plésiat P. Role of the MexEF-OprN efflux system in low-level resistance of Pseudomonas aeruginosa to ciprofloxacin. Antimicrob Agents Chemother 2011;55:5676–84. https://doi.org/10.1128/AAC.00101-11.

[18] Sobel ML, Neshat S, Poole K. Mutations in PA2491 (mexS) promote MexT-dependent mexEF-oprN expression and multidrug resistance in a clinical strain of Pseudomonas aeruginosa. J Bacteriol 2005;187:1246–53. https://doi.org/10.1128/JB.187.4.1246-1253.2005.

[19] Cabot G, Ocampo-Sosa AA, Domínguez MA, Gago JF, Juan C, Tubau F, et al. Genetic markers of widespread extensively drug-resistant Pseudomonas aeruginosa high-risk clones. Antimicrob Agents Chemother 2012;56:6349–57. https://doi.org/10.1128/AAC.01388-12.

[20] Caille O, Zincke D, Merighi M, Balasubramanian D, Kumari H, Kong K-F, et al. Structural and functional characterization of Pseudomonas aeruginosa global regulator AmpR. J Bacteriol 2014;196:3890–902. https://doi.org/10.1128/JB.01997-14.

[21] Sirgel FA, Tait M, Warren RM, Streicher EM, Böttger EC, van Helden PD, et al. Mutations in the rrs A1401G Gene and Phenotypic Resistance to Amikacin and Capreomycin in Mycobacterium tuberculosis. Microb Drug Resist 2012;18:193–7. https://doi.org/10.1089/mdr.2011.0063.

[22] Maus CE, Plikaytis BB, Shinnick TM. Molecular Analysis of Cross-Resistance to Capreomycin, Kanamycin, Amikacin, and Viomycin in Mycobacterium tuberculosis. Antimicrob Agents Chemother 2005;49:3192–7. https://doi.org/10.1128/AAC.49.8.3192-3197.2005.

[23] Nessar R, Reyrat JM, Murray A, Gicquel B. Genetic analysis of new 16S rRNA mutations conferring aminoglycoside resistance in Mycobacterium abscessus. J Antimicrob Chemother 2011;66:1719–24. https://doi.org/10.1093/jac/dkr209.

[24] Springer B, Kidan YG, Prammananan T, Ellrott K, Bottger EC, Sander P. Mechanisms of Streptomycin Resistance: Selection of Mutations in the 16S rRNA Gene Conferring Resistance. Antimicrob Agents Chemother 2001;45:2877–84. https://doi.org/10.1128/AAC.45.10.2877-2884.2001.

[25] Lau CH-F, Krahn T, Gilmour C, Mullen E, Poole K. AmgRS-mediated envelope stress-inducible expression of the mexXY multidrug efflux operon of Pseudomonas aeruginosa. Microbiologyopen 2015;4:121–35. https://doi.org/10.1002/mbo3.226.

[26] Lau CH-F, Fraud S, Jones M, Peterson SN, Poole K. Mutational Activation of the AmgRS Two-Component System in Aminoglycoside-Resistant Pseudomonas aeruginosa. Antimicrob Agents Chemother 2013;57:2243–51. https://doi.org/10.1128/AAC.00170-13.

[27] Schniederjans M, Koska M, Häussler S. Transcriptional and Mutational Profiling of an Aminoglycoside-Resistant Pseudomonas aeruginosa Small-Colony Variant. Antimicrob Agents Chemother 2017;61. https://doi.org/10.1128/AAC.01178-17.

[28] Moskowitz SM, Ernst RK, Miller SI. PmrAB, a two-component regulatory system of Pseudomonas aeruginosa that modulates resistance to cationic antimicrobial peptides and addition of aminoarabinose to lipid A. J Bacteriol 2004;186:575–9. https://doi.org/10.1128/jb.186.2.575-579.2004.

[29] Bolard A, Plésiat P, Jeannot K. Mutations in Gene fusA1 as a Novel Mechanism of Aminoglycoside Resistance in Clinical Strains of Pseudomonas aeruginosa. Antimicrob Agents Chemother 2018;62:e01835-17. https://doi.org/10.1128/AAC.01835-17.

[30] López-Causapé C, Rubio R, Cabot G, Oliver A. Evolution of the Pseudomonas aeruginosa Aminoglycoside Mutational Resistome In Vitro and in the Cystic Fibrosis Setting. Antimicrob Agents Chemother 2018;62:e02583-17. https://doi.org/10.1128/AAC.02583-17.

[31] Islam S, Oh H, Jalal S, Karpati F, Ciofu O, Høiby N, et al. Chromosomal mechanisms of aminoglycoside resistance in Pseudomonas aeruginosa isolates from cystic fibrosis patients. Clin Microbiol Infect 2009;15:60–6. https://doi.org/10.1111/j.1469-0691.2008.02097.x.

[32] Poonsuk K, Tribuddharat C, Chuanchuen R. Aminoglycoside resistance mechanisms in Pseudomonas aeruginosa isolates from non-cystic fibrosis patients in Thailand. Can J Microbiol 2013;59:51–6. https://doi.org/10.1139/cjm-2012-0465.
